# Low circulatory levels of total cholesterol, HDL-C and LDL-C are associated with death of patients with sepsis and critical illness: systematic review, meta-analysis, and perspective of observational studies

**DOI:** 10.1016/j.ebiom.2024.104981

**Published:** 2024-01-29

**Authors:** Rory Taylor, Chengyuan Zhang, Deslit George, Sarah Kotecha, Mariam Abdelghaffar, Thorsten Forster, Patricia Dos Santos Rodrigues, Alexander C. Reisinger, Daniel White, Fergus Hamilton, W. John Watkins, David M. Griffith, Peter Ghazal

**Affiliations:** a Deanery of Biomedical Sciences, University of Edinburgh Medical School, Edinburgh, UK; b Department of Anaesthesia, Critical Care and Pain Medicine, NHS Lothian, Edinburgh, UK; c School of Medicine, University of Cardiff, Cardiff, UK; d Department of Child Health, School of Medicine, University of Cardiff, Cardiff, UK; e School of Medicine, Royal College of Surgeons in Ireland, Bahrain; f LifeArc, Edinburgh Bioquarter, Edinburgh, UK; g Department of Internal Medicine, Intensive Care Unit, Medical University of Graz, Graz, Austria; h Project Sepsis, Systems Immunity Research Institute, School of Medicine, University of Cardiff, Cardiff, UK; i MRC Integrative Epidemiology Unit, University of Bristol, Bristol, UK; j Dept of Immunity and Infection, School of Medicine, Cardiff University, Cardiff, UK; k Anaesthesia, Critical Care and Pain, Molecular, Genetics, and Population Health Sciences, University of Edinburgh, Edinburgh, UK

**Keywords:** Sepsis, Critical illness, Lipids, Cholesterol, Immunometabolism

## Abstract

**Background:**

Mechanistic studies have established a biological role of sterol metabolism in infection and immunity with clinical data linking deranged cholesterol metabolism during sepsis with poorer outcomes. In this systematic review we assess the relationship between biomarkers of cholesterol homeostasis and mortality in critical illness.

**Methods:**

We identified articles by searching a total of seven electronic databases from inception to October 2023. Prospective observational cohort studies included those subjects who had systemic cholesterol (Total Cholesterol (TC), HDL-C or LDL-C) levels assessed on the first day of ICU admission and short-term mortality recorded. Meta-analysis and meta-regression were used to evaluate overall mean differences in serum cholesterol levels between survivors and non-survivors. Study quality was assessed using the Newcastle–Ottawa Scale.

**Findings:**

From 6469 studies identified by searches, 24 studies with 2542 participants were included in meta-analysis. Non-survivors had distinctly lower HDL-C at ICU admission −7.06 mg/dL (95% CI −9.21 to −4.91, p < 0.0001) in comparison with survivors. Corresponding differences were also seen less robustly for TC −21.86 mg/dL (95% CI −31.23 to −12.49, p < 0.0001) and LDL-C −8.79 mg/dL (95% CI, −13.74 to −3.83, p = 0.0005).

**Interpretation:**

Systemic cholesterol levels (TC, HDL-C and LDL-C) on admission to critical care are inversely related to mortality. This finding is consistent with the notion that inflammatory and metabolic setpoints are coupled, such that the maladaptive-setpoint changes of cholesterol in critical illness are related to underlying inflammatory processes. We highlight the potential of HDL-biomarkers as early predictors of severity of illness and emphasise that future research should consider the metabolic and functional heterogeneity of HDLs.

**Funding:**

EU-ERDF-Welsh Government Ser Cymru programme, 10.13039/501100000268BBSRC, and EU-FP7 ClouDx-i project (PG).

**Research in context**

**Evidence before this study**

Existing mechanistic studies have linked sterol metabolism with immune and inflammatory functions. Observational clinical data indicates that there are alterations of serum lipids, most notably serum cholesterol and high-density lipoprotein cholesterol, during sepsis and critical illness and that the degree of this change is related to adverse outcomes. Existing systematic reviews and meta-analyses of observational studies support this observation within sepsis cohorts.

**Added value of this study**

Our study further confirms the inverse association between hypocholesterolaemia and critical illness and mortality. We provide an increased sample size compared to previous systematic reviews and extend our observations to general critical illness rather than just being limited to sepsis cohorts. We discuss plausible biological connectivity with immunometabolism and provide a perspective of more recently published epidemiological biobank studies examining pre-existing lipoprotein particles sizes and the risk of death from sepsis.

**Implications of all the available evidence**

Our findings indicate that lowered HDL-C, LDL-C and TC levels either prior to or early during sepsis and critical illness is adversely prognostic. Hence a hallmark feature of sepsis and critical illness is low systemic levels of cholesterol and its associated lipoproteins (with HDL-C showing the highest reliability). We propose HDL-biomarkers may have potential as early predictors of severity of illness but emphasise that the association with HDL-cholesterol total quantity may not be causal. Future prognostic and therapeutic studies need to consider the metabolic and functional heterogeneity of HDLs when examining this relationship.

## Introduction

Altered levels in concentration of serum total cholesterol (TC), high-density lipoprotein cholesterol (HDL-C) and low-density lipoprotein cholesterol (LDL-C) from the expected normal equilibrium range are recognised features associated with *chronic* inflammatory conditions.^1^, ^2^, ^3^

Biologically, there is an historical and growing base of evidence at multiple levels that the cholesterol metabolic pathway including its metabolites, serum cholesterol and lipoproteins are interlinked with inflammation and infection. On an immunometabolism level, cholesterol metabolism has central and cross-regulatory roles in immunity and host-defense where cholesterol metabolism has been shown to be directly regulated by the interferon cytokine response, with upstream and downstream metabolites of the pathway directing immune effector functions and anti-infective activity.^4^^,^^5^ On a lipoprotein level, HDL appears to neutralise and promote clearance of toxins from infections in both Gram-negative bacteria (Lipopolysaccharide, LPS) and Gram-positive bacteria (lipoteichoic acid, LTA).^6^, ^7^, ^8^, ^9^ Non-neutralised levels of LPS and LTA are recognised and initiate an inflammatory cascade through Toll-Like pattern recognition receptors (TLR) TLR4 and TLR2/6, respectively, and this cascade has been proposed to be curtailed by HDL through the anti-inflammatory Activating Transcription Factor 3 (ATF3) pathway.^10^, ^11^, ^12^ Thus, in the biological context of an acute infection-inflammatory response changes in levels of cholesterol metabolism and homeostasis would be anticipated.

Clinically, key inflammatory and anti-inflammatory cytokines such as Tumour Necrosis Factor α (TNFα), Interleukin 6 (IL-6) and Interleukin 10 (IL-10) are clearly elevated during and associated with the severity of sepsis^13^, ^14^, ^15^, ^16^ but most notably have an inverse correlation with serum cholesterol levels in patients with sepsis and septic shock.^17^, ^18^, ^19^ In addition, there are numerous clinical observational studies reporting inverse associations between serum cholesterol levels and mortality in critically ill adults, mainly focused on sepsis.^17^^,^^19^, ^20^, ^21^, ^22^ Although limited in population size and scope, these studies consistently report associations between low levels of cholesterol (HDL-C, LDL-C and TC) and mortality in sepsis.^23^^,^^24^ However, at present there is a lack of clinical trials examining the diagnostic or prognostic utility of serum TC or its associated lipoproteins. By contrast, HDL-based infusion is currently being explored as a potential therapy for sepsis^7^^,^^25^^,^^26^ even though a causative relationship between serum cholesterol and outcome remains to be established.

The question we seek to address is whether maladaptive-setpoint changes (described by the concentration of key constituents) in cholesterol lipoprotein pathways represents a hallmark feature of *acutely severe* inflammatory states such as in sepsis and critical illness. Therefore, we believe a comprehensive appraisal and meta-analysis of systemic levels of LDL-C, HDL-C, total cholesterol, and mortality across mixed (sepsis and non-sepsis) intensive care unit (ICU) populations is timely. Here, we quantitatively review the evidence for a relationship between serum cholesterol (HDL-C, LDL-C, and TC) and death (<30 days) in critical illness. We further provide a perspective of our findings together with emerging large scale population studies on antecedent lipoprotein levels, notably HDL particle levels and risk of sepsis, highlighting areas for future diagnostic and therapeutic research.

## Methods

This systematic review was reported according to the recommendations of the Meta-analysis of Observational Studies in Epidemiology (MOOSE) guidelines (Supplementary Table S1).^27^ The protocol was registered in PROSPERO, registration number CRD42016052809.

### Search strategy

Embase, Ovid Medline, Cumulative Index to Nursing and Allied Health Literature (CINAHL), Cochrane Central Register of Controlled Trials, Web of Science, Pubmed and SCOPUS databases were searched from inception to 03 October 2023, to identify prospective observational studies which investigated associations between serum cholesterol (total cholesterol and/or HDL-C and/or LDL-C) on day of ICU admission and mortality in patients with critical illness. Reference lists of included studies were hand-searched to identify additional studies. An attempt was made to contact all authors via the listed correspondence email address to receive patient level data and no unpublished studies were used. A combination of key word search terms related to sepsis, cholesterol and intensive care were combined with relevant MeSH terms to identify potentially relevant articles from databases. Full search strategies and logic are available in Supplementary material.

### Study selection

Duplicates were removed and articles were screened for potential relevance based on their title and abstracts. Full texts of relevant studies were retrieved and assessed for suitability of inclusion by whether they met inclusion and exclusion criteria, conference abstracts with no full-text available were not included. Inclusion criteria were: [1] prospective study, [2] adult human subjects (≥16 years of age), [3] intensive care population, [4] in-hospital, ICU or short-term mortality (<30 day) recorded, [5] serum total cholesterol and/or HDL-cholesterol and/or LDL-cholesterol measured on day of ICU admission, [6] authors measured an association between serum cholesterol and hospital mortality. Exclusion criteria were: [1] interventional study assessing efficacy and/or safety of a therapeutic intervention in addition to standard of care. Non-English articles were considered if full text was available. The database search and study selection process were carried out by two independent reviewers (RT, CZ) with any discrepancies resolved by discussion and mutual consensus.

### Data extraction

The following data were extracted from the final list of articles to enable assessment of whether serum cholesterol was associated with mortality in prospective observational ICU cohorts: [1] first author surname, [2] year of publication, [3] country and setting where study was performed, [4] study design and characteristics, [5] number of participants, [6] inclusion and exclusion criteria of study, [7] participant age and sex, [8] serum cholesterol mg/dL or mmol/L (total, LDL-C or HDL-C) assessed within 24 h of ICU admission, [9] in-hospital mortality, ICU mortality or 28/30 day mortality recorded, [10] key results relating to serum cholesterol and mortality.

### Risk of bias assessment

A modified version of the Newcastle–Ottawa Scale (NOS) for cohort studies was used to assess study quality.^28^ As part of this process, we identified potential confounders of the relationship between HDL-C, LDL-C and total cholesterol and mortality. We reviewed each paper for evidence that the authors measured and/or adjusted for these variables (Supplementary Table S2).

### Statistics (meta-analysis and meta-regression)

Unadjusted serum cholesterol concentration on the first day of ICU admission was reported by included studies as either mean (standard deviation) or median (interquartile range). We converted median and interquartile range to mean and standard deviation using previously outlined methods.^29^ To compare risk factors across non-survivor and survivor study groups we combined data between studies for each of three independent variables: Total cholesterol (22 studies, n = 2419), LDL cholesterol (16 studies, n = 1386), and HDL cholesterol (17 studies, n = 1542). Mean difference (95% CI) between surviving and non-surviving patients was calculated for each independent variable. Unadjusted serum cholesterol levels as reported in studies were used for this analysis. When presented in mmol/L serum cholesterol levels were converted to mg/dL using a multiplication factor of 38.66976. All meta-analyses estimated overall effects under a Random Effects Model. We did not assess for skewness in our meta-analysis due to a lack of availability of individual patient data in the included studies. Heterogeneity was calculated using the I^2^ statistic and I^2^ values greater than 50% and 75% were considered to indicate moderate and high heterogeneity, respectively. An effect size p-value of <0.05 for the t-distribution in the meta-analyses and meta-regressions was considered significant. Publication bias was assessed visually using funnel plots and also using Egger's regression test to quantify asymmetry with p-value <0.05 as the threshold for statistical significance. Additionally, a meta-regression was conducted for all three variables (TC, HDL-C, LDL-C) to allow adjustment for age and the proportion of males in each study that had this data available, with the adjusted forest plots produced. All meta-analyses and regressions were conducted using R package Metafor 3.8-1.

### Role of funders

The funders had no role in the design of the study; the collection, analysis, and interpretation of data; or approval of the final manuscript.

### Ethics

Ethics committee approval was not required for this systematic review and meta-analysis of anonymous publicly available datasets.

## Results

### Study selection

**Fig. 1:**
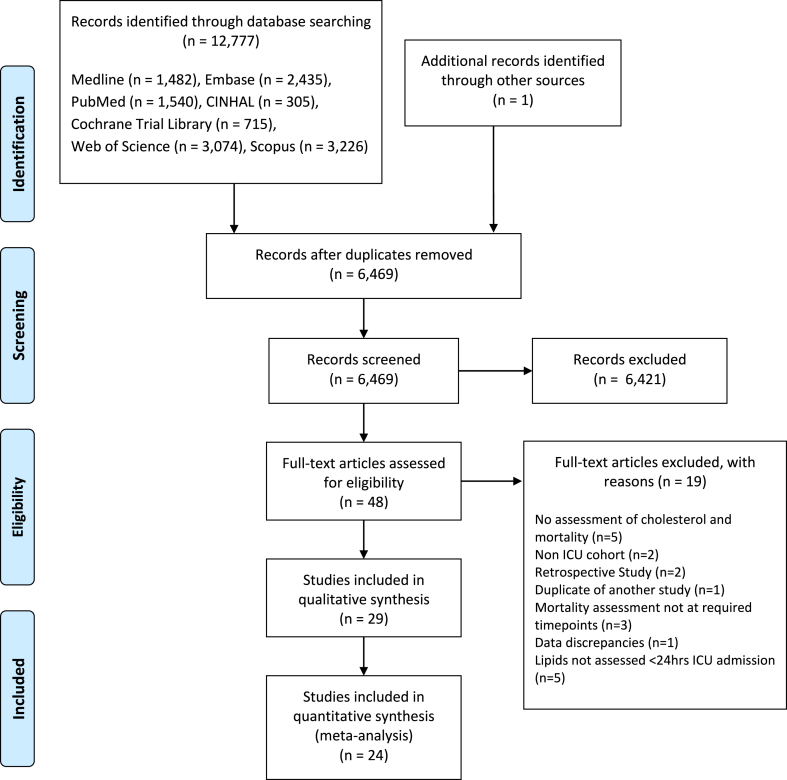
Study selection flow-diagram showing included and excluded studies at each stage.

Fig. 1 shows a flow diagram of database searches. The search (Medline, Embase, CINAHL, Cochrane Central Register of Controlled Trials Database, Pubmed, Scopus, Web of Science from inception to 03 Oct 2023) identified 12,777 records. One additional record was identified from handsearching the references of included studies.^30^ After duplicates were removed there was 6469 unique records. The titles and abstracts of these records were screened for potential inclusion. This led to 48 full text articles being assessed for eligibility, with 29 articles included in systematic review. Twenty-four studies were included in quantitative meta-analysis, primarily using numerical data from manuscripts and supplemental data, with one study included using unpublished mortality stratified data obtained from contacting the authors.^31^ Our study selection process is outlined in Fig. 1 and excluded studies with reasons for exclusion are summarised in Supplementary Table S3.

### Study characteristics

A total of 29 studies published between 2000 and 2022 were included in the review^17^^,^^19^, ^20^, ^21^, ^22^^,^^30^, ^31^, ^32^, ^33^, ^34^, ^35^, ^36^, ^37^, ^38^, ^39^, ^40^, ^41^, ^42^, ^43^, ^44^, ^45^, ^46^, ^47^, ^48^, ^49^, ^50^, ^51^, ^52^, ^53^ (Table 1). All studies were prospective observational cohort studies with mixed sex adult participants. There was a total of 3003 participants across all 29 studies with study size ranging from 17 to 502. Age of participants were reported in all but one study^30^ as either median or mean age and this ranged from 34 to 75. Twenty eight of the studies assessed serum total cholesterol on day of admission to critical care,^17^^,^^19^, ^20^, ^21^, ^22^^,^^30^, ^31^, ^32^, ^33^, ^34^, ^35^, ^36^, ^37^, ^38^, ^39^^,^^41^, ^42^, ^43^, ^44^, ^45^, ^46^, ^47^, ^48^, ^49^, ^50^, ^51^, ^52^, ^53^ twenty two assessed HDL cholesterol^17^^,^^19^^,^^20^^,^^22^^,^^31^, ^32^, ^33^, ^34^^,^^36^, ^37^, ^38^^,^^40^, ^41^, ^42^, ^43^^,^^45^, ^46^, ^47^, ^48^, ^49^, ^50^, ^51^ and 20 assessed LDL cholesterol.^17^^,^^19^^,^^20^^,^^22^^,^^32^, ^33^, ^34^^,^^36^, ^37^, ^38^^,^^41^^,^^43^^,^^45^, ^46^, ^47^, ^48^, ^49^, ^50^, ^51^ Characteristics of included studies are shown in Table 1. Twenty-four of these studies were synthesised in quantitative meta-analysis with 2542 participants.^17^^,^^20^, ^21^, ^22^^,^^30^, ^31^, ^32^^,^^34^^,^^36^^,^^37^^,^^39^, ^40^, ^41^^,^^43^, ^44^, ^45^, ^46^, ^47^, ^48^, ^49^, ^50^, ^51^, ^52^, ^53^ All studies assessed patients admitted to critical care. One study^51^ assessed patients with critical illness and sepsis in the emergency department with a high proportion (>90%) admitted directly to the ICU. As patient level data was available, we were able to analyse the cohort who were admitted to ICU as part of our meta-analysis. Thirteen of the 24 studies included in the meta-analysis recruited patients with a diagnosis of sepsis on ICU admission.^17^^,^^20^^,^^21^^,^^34^^,^^40^^,^^41^^,^^44^^,^^46^^,^^48^, ^49^, ^50^, ^51^^,^^53^ The remaining studies included in the meta-analysis assessed participants with: acute kidney injury,^39^ acute liver failure,^32^ acute heart failure,^22^ severe acute pancreatitis,^43^ multiple organ failure,^30^ systemic inflammatory response syndrome (SIRS),^36^ surgical ICU admissions,^47^ a mixture of patients with sepsis and non-septic controls,^31^ general ICU cohort,^52^ severe community acquired pneumonia^37^ and COVID-19 pneumonia.^45^

**Table 1:** Shows characteristics of included studies, listed by name of lead author, TC = total cholesterol.

Study	Setting (region, setting, dates)	Number of participants (survivor; nonsurvivor)	Sex (male) (% male)	Patient groups (subgroup)	Age (years) (mean or median)	Exclusion criteria	Cholesterol biomarkers assessed	Key results relating to Cholesterol biomarkers
Barati et al., 2011	Iran, Hospital ICU, Mar 2009–Feb 2010	70 (40; 30)	28/70 (40%)	Sepsis, non-sepsis	72 ± 15 (mean ± SD)	Trauma or post-surgical patients	TC, HDL-C, LDL-C	In Sepsis group TC of non-survivors was higher than survivors (101.6 ± 37.5 vs 69.4 ± 8.3) (p < 0.001) on admission to ICU. No significant differences for mortality for LDL-C and HDL-C.
Barlage et al., 2009	Germany, Hospital ICU	151 (104; 47)	108/151 (72%)	Sepsis	Survivors 59 (41–67) (median, IQR) Non-survivors 60 (44–73) (median, IQR)	HIV/leukemia, transplant recipients, receiving chemotherapy or high dose corticosteroids	TC, HDL-C, LDL-C	TC, HDL-C and LDL-C lower in non-survivors than survivor on day 1 (TC, p = 0.017) (LDL-C, p = 0.012) (HDL-C, p = 0.049)
Berbee et al., 2008	Netherlands, Hospital ICU, Nov 1997–Jan 2000	17 (9; 8)	9/17 (53%)	Severe sepsis	Survivors 62.0 (51.0–71.0) (median, IQR) Non-survivors 68.3 (66.3–70.0) (median, IQR)	Patient could not receive coumarin derivates, heparin in anticoagulant doses, or NSAIDs in anti-inflammatory doses	TC, HDL-C, LDL-C	No significant differences in TC, HDL-C and LDL-C survivors vs non-survivors.
Biller et al., 2014	Germany, Hospital ICU, 2007–2011	116 (79; 37)	77/116 (66%)	Infected, Non-infected	Infected group 69.5 (25–96) (median, range) Non Infected 58.3 (22–84) (median, range)		TC	Infected group non-survivors had lower TC than survivors on day1 (p = 0.006). No significant difference in non infected group.
Bonville et al., 2004	USA, Hospital surgical ICU	109 (82; 27)	59/109 (54%)	Systemic Inflammatory Response Syndrome (SIRS)	Survivors 63.7 ± 1.9 (mean ± SD) Non-survivors 69.9 ± 2.9 (mean ± SD)		TC, HDL-C, LDL-C	No significant differences between survivors and non-survivors on day 1, TC fell significantly from day 1 to day 2 in non survivor group (p = 0.0066) and persisted until day 7.
Chien et al., 2005	Taiwan, Hospital ICU, Oct 2002–Jan 2003	63 (44; 19)	36/63 (57%)	Severe sepsis	Survivors 72.5 (18–92) (median, range) Non-survivors 72 (23–87) (median, range)	Cirrhosis, terminal cancer, history of hyperlipidemia, receiving TPN	TC, HDL-C, LDL-C	HDL-C significantly lower in non-survivors than survivors from day 1–4.
Chien et al., 2015	Taiwan, Hospital ICU, Nov 2006–Jan 2009	40 (25; 15)	27/40 (67%)	Severe community acquired pneumonia	Survivors 74.4 ± 13.7 (mean ± SD) Non-survivors 76.3 ± 14.8 (mean ± SD)	Previous hospital admission in last 40 days, pregnancy, long term mechanical ventilator dependence, severe immunosuppression: HIV/AIDS, organ transplant, receiving chemotherapy	TC, HDL-C, LDL-C	HDL-C and LDL-C significantly lower on day 7 in non survivors vs survivors, no significant difference on day of admission.
Delirrad et al., 2020	Iran, ICU, March 2017–March 2018	88 (32; 56)	48/88 (55%)	Sepsis	Survivors 67.62 ± 11.24 (mean ± SD) Non-survivors 73.70 ± 10.43 (mean ± SD)	Lipid reducing medications	TC, LDL-C, HDL-C	TG was significantly higher in non-survivors TC, HDL-C, LDL-C did not show statistical significance between both groups
Desmoulin et al., 2013	France, Hospital Cardiac ICU, Jul 2008–Apr 2009	126 (98; 28)	77/126 (61%)	Acute heart failure	Survivors 69 ± 15 (mean ± SD) Non-survivors 69 ± 13 (mean ± SD)	Stroke in last 6 months, Acute/chronic liver failure, History of drug/alcohol abuse, cancer in last 5 years, haematological pathology, severe neutropenia	TC, HDL-C, LDL-C	HDL-C and LDL-C on day 1 were lower in non survivors than survivors. (HDL-C, p = 0.005), (LDL-C, p = 0.046). A low level of HDL-C was a significant predictor of 30 day mortality.
Etogo-Asse et al. 2012	London, Hospital ICU, Jan 2007–Aug 2009	163 (64; 99)	93/163 (57%)	Acute liver failure (Acute on chronic; acute)	42 [30–54] (median)[IQR]	Post operative care, therapy with etomidate, corticosteroid/lipid modulating therapy <3 months of admission, parenteral nutrition	TC, HDL-C, LDL-C	On day 1 HDL-C significantly lower in non-survivors with AOCLF and ALF (p < 0.001), TC lower in non-survivors ALF group (p < 0.05).
Fraunberger et al., 2000	Germany, Hospital ICU	33		Multi-organ failure			TC	TC lower in non-survivors than survivors on admission to study. (p < 0.05)
Gordon et al., 2001	New York, Surgical ICU	111 (102; 9)	61/111 (55%)	Surgical patient	65 (19–97) (mean, range)		TC, HDL-C, LDL-C	Group with TC level <120 mg/dL more likely to have length of stay >2days (p = 0.006)
Guimaraes et al., 2008	Brazil, ICU	56 (17; 39)	36/56 (64%)	Acute kidney injury	Survivors 57.5 ± 17.3 (mean ± SD) Non-survivors 59.5 ± 13.9 (mean ± SD)	Pre-existing renal insufficiency, ICU stay <48 h, urinary tract obstruction induced AKI, elevated serum creatinine resolved within 48 h	TC	TC lower in non-survivors than survivors on day 1 (p < 0.001)
Guirgis et al., 2018	USA, ICU/Emergency department	84^a^^b^ (67; 17)	43/84 (51%)	Sepsis	64 ± 14 (mean ± SD)	1) pregnancy, 2) lack of valid consent, 3) familial or genetic disorders of lipid metabolism, 4) active seizure, and 5) cardiopulmonary resuscitation prior to enrollment.	TC, HDL-C, LDL-C	Dys-HDL levels were significantly associated with in-hospital death. Early cholesterol levels significantly associated with severity of organ failure and death from sepsis.
Inal et al., 2015	Turkey, Hospital ICU,	85 (46; 39)	48/85 (56%)	Sepsis	65.2 ± 17.9 (mean ± SD)	End stage cancer, liver cirrhosis, hyperlipidemia, receiving lipid lowering drugs or total parenteral nutrition	HDL-C	HDL-C significantly lower in non-survivors than survivors on day 1 (p < 0.001).
Khaliq et al., 2020	United Kingdom, Hospital ICU Jan 2014–Apr 2015	19 (11; 8)	16/19 (84%)	Sepsis	Survivor 67.4 ± 16.3 (mean ± SD) Non-survivor 68.5 ± 12.6 (mean ± SD)	Age <18 years old, pregnant, severe psychiatric illness, cirrhosis, severe brain injury, >24 h hospital admission prior to ICU admission, patients using cortisol lower drugs within preceding 6 weeks,	TC, HDL-C, LDL-C	Survivors had higher HDL-C and LDL-C than non survivors.
Lee et al., 2015	South Korea, Hospital ICU, Aug–Dec 2008	117 (65; 52)	73/115 (63%)	Sepsis	62.7 ± 16.2 (mean ± SD)	Under 18, Pregnant, Readmission to ICU, need for primary cardiac care, liver disease/hepatitis, dyslipidaemia, steroid/statin use in last 7 days	TC, HDL-C, LDL-C	No significant difference in TC, HDL-C, LDL-C between survivors and non-survivors on day 1, an elevated LDL-C on day 1 was associated with poor prognosis
Lekkou et al., 2014	Greece, Hospital ICU	50 (28; 22)	27/50 (54%)	Severe sepsis	Survivors 64.8 ± 3.2 (mean ± SD) Non-survivors 66.2 ± 6.5 (mean ± SD)	Trauma, HIV, neutropenia, endstage hepatic or renal disease, hyperlipidemia	TC, HDL-C, LDL-C	Day 1 HDL-C lower in non-survivors, Day 3 TC and HDL-C were lower in non-survivors, Day 10 TC, HDL-C, LDL-C were lower in non-survivors
Levels et al., 2007	Netherlands, Hospital ICU	26 (15; 11)		Severe sepsis	Survivors 60 ± 3 (mean ± SD) Non-survivors 60 ± 4 (mean ± SD)	<18 years old, organ transplant recipient, uncontrolled hemorrhage, cardiogenic shock, burns as primary admission reason	TC, HDL-C, LDL-C	Admission total cholesterol and LDL-C were significantly lower in non-survivors than survivors
Luthold et al., 2007	Switzerland, Medical ICU, Sep 1996–Jun 1997	101 (78; 23)	55/101 (54%)	ICU admission	59 (23–86) (median, range)		TC, HDL-C	No significant differences in TC and HDL-C between survivor/non-survivors on day of admission.
Memis et al., 2007	Turkey, Hospital ICU	96 (55; 41)	40/96 (42%)	Severe sepsis	56 (18–75) (median, range)	Burns, Trauma, receiving coronary care, immunosuppressed patients, neutropenia	TC	TC significantly lower in non-survivors than survivors on day 1 (92.2 ± 25.1 vs 175.1 ± 38.6) (p < 0.001)
Oliva et al., 2022	Cuba, Hospital ICU, Jan 2018–Jan 2019	224 (132; 92)		Sepsis	Survivors 53 ± 21 (mean ± SD) Non survivors 65 ± 15 (mean ± SD)	Transferred from another institution	TC	TC significantly lower in non survivors than survivors on day of admission to ICU (p = 0.001)
Peng et al., 2015	Taiwan, ICU, Feb 2008–Sep 2011	66 (53; 13)	38/66 (58%)	Predicted severe acute pancreatitis	Survivors 60 (45–76.5) (median, IQR) Non-survivors 59 (45–84) (median, IQR)	Prior history of acute pancreatitis or lipid lowering agents, receiving total parenteral nutrition	TC, HDL-C, LDL-C	HDL-C on admission higher in survivors than non-survivors (p < 0.001)
Reisinger et al., 2020	Austria, Hospital ICU, 2017–2018	78 (55; 23)	42/78 (54%)	Sepsis cohort and Non-sepsis control cohort	Sepsis 66 (50–75) (median, IQR) Non-sepsis 72 (65–79) (median, IQR)	Palliative care, comfort terminal care, AIDS, pregnancy, Age >100 years	TC, HDL-C	TC and HDL not associated with 28 day mortality. Dyslipidemia is more common is sepsis than non sepsis controls. HDL functionality measured by arylesterase activity of paraoxonase associated with mortality
Tachyla et al., 2017	Belarus, ICU, 2010–2015.	67 (38; 29)	39/67 (58%)	Sepsis and Multiple organ dysfunction	Survivors 46.0 (40–61) (median, IQR) Non-survivors 62.5 (56–75) (median, IQR)	Chronic pre-existing organ failure, immunodeficiency, steroid treatment, lipid lowering therapy, AIDS, metastatic cancer, haematological malignancy	TC	No significant difference in TC between both groups on Day 1 Day 5 TC decreased in both groups. Last day TC was lower in non-survivors (p = 0.016)
Tanaka et al., 2017	France, Surgical ICU, July–Sep 2014.	75 (63; 12)	53/75 (71%)	Sepsis, Trauma	62 (42–74) (median, IQR)	Liver cirrhosis, immunocompromised (acquired immune deficiency, neutropenia, transplant)	TC, HDL-C, LDL-C	Lipid levels in sepsis group not associated with mortality. Poor outcome defined as death or SOFA score >6 at day 3 was associated with lower HDL-C levels (p = 0.03).
Tanaka et al., 2020	France, Hospital ICU, Mar 2020–April 2020	48 (32; 16)	31/48 (65%)	Severe COVID-19 cohort	Survivors 55 (45–62) (median, IQR) Non-survivors 59 (50–67) (median, IQR)		TC, HDL-C, LDL-C	HDL-C and LDL-C on ICU admission were not associated with poor outcomes in Covid19 pneumonia patient. However, low lipoprotein concentrations were associated with mortality in cases with bacterial superinfection during ICU stay, reinforcing a potential role of these particles during bacterial sepsis.
Tanaka et al., 2022	France, Hospital ICU, May 2016 to Apr 2020	226^b^ (184; 42)	121/226 (54%)	Sepsis	63 (52–72) (median, IQR)	Pre-existing liver disease, immunocompromised patients (AIDS or transplant surgery)	TC, HDL-C, LDL-C	Day 28 mortality was significantly higher with low TC, HDL-C and LDL-C at admission.
Uzundere et al., 2015	Turkey, Hospital ICU, Mar 2012–Mar 2013	502 (330; 172)	283/502 (56%)	General ICU cohort	Survivors 58.5 ± 18.5 (mean ± SD) Non survivors 66.6 ± 15.3 (mean ± SD)	Patients <18 years old, pregnant, brain death, <24 h stay in ICU, chronic renal disease	TC	TC was significantly lower in non survivors than survivors on day of admission (p = 0.032)

HDL-C = high-density lipoprotein cholesterol. LDL-C = low-density lipoprotein cholesterol.

a Guirgis et al. was a cohort of patients with critical illness and sepsis in ED, with a very high proportion of patients admitted directly to ICU (>80%). Using patient level data from the study we included only the septic cohort who were admitted directly to ICU, these are the numbers listed.

b These studies were included in the meta-analysis on the basis of patient level data from supplementary files, subject numbers will vary slightly between this table and the cohort numbers in the meta-analysis due to missing data values in these supplementary files.

### Risk of bias assessment

Study quality ranged from 5 to 7 out of a total possible score of 9 (Supplementary Table S2). The greatest area of potential bias which was uniform across all studies, was the poor comparability of the survivor and non-survivor cohorts as a result of undetected (and/or unadjusted) confounding factors which were unaccounted for. Several studies reported on important baseline confounding factors such as age, body mass index (BMI) and co-morbidity, and performed some degree of multivariable regression analysis, however only six performed multivariable regression analysis using demographic and/or co-morbidity factors.^20^^,^^31^^,^^32^^,^^38^^,^^41^^,^^53^ These studies also failed to account for all the important confounding factors which we identified in our modification of the NOS tool (Supplementary Table S2).

### Meta-analysis

Data from 24 studies with 2542 patients were pooled in quantitative meta-analyses.^17^^,^^20^, ^21^, ^22^^,^^30^, ^31^, ^32^^,^^34^^,^^36^^,^^37^^,^^39^, ^40^, ^41^^,^^43^, ^44^, ^45^, ^46^, ^47^, ^48^, ^49^, ^50^, ^51^, ^52^, ^53^ Pooled weighted mean differences were used to quantify differences between serum total, HDL cholesterol and LDL cholesterol levels in survivors and non-survivors.

#### Total cholesterol

**Fig. 2:**
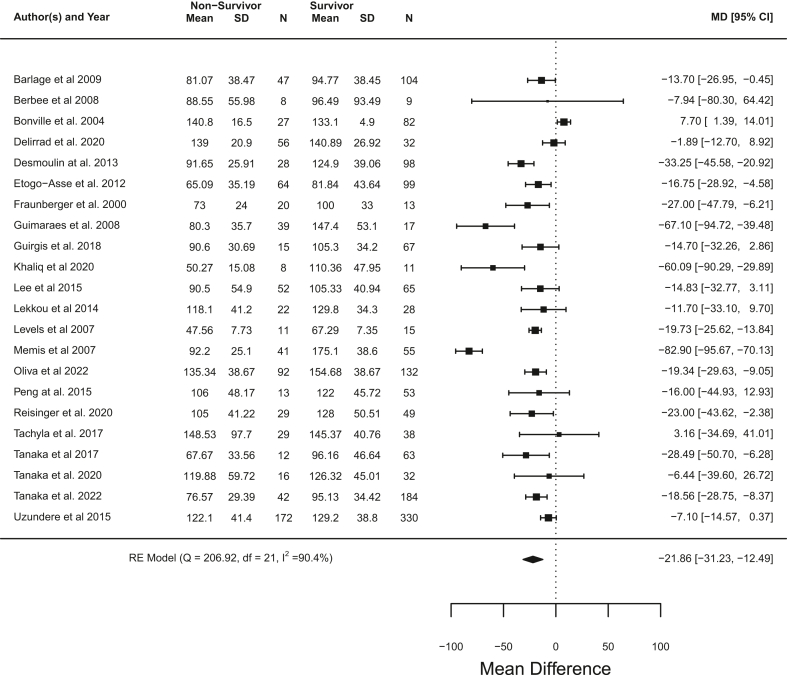
Unadjusted meta-analysis for total cholesterol showing pooled mean difference in serum total cholesterol and 95% CI between non-survivors and survivors (control) across studies (mg/dL). Heterogeneity shown as I^2^.

Participants that died had significantly lower serum total cholesterol on day 1 of ICU admission when compared to survivors, with a pooled mean difference of −21.86 mg/dL (95% CI −31.23 to −12.49) (p < 0.0001, meta analysis t-distribution) across 22 studies with 2419 participant^17^^,^^20^, ^21^, ^22^^,^^30^, ^31^, ^32^^,^^34^^,^^36^^,^^39^^,^^43^, ^44^, ^45^, ^46^, ^47^, ^48^, ^49^, ^50^, ^51^, ^52^, ^53^, ^54^ (Fig. 2). A high level of heterogeneity was present (I^2^ = 90.4%). Adjusting the meta-analysis for mean age and proportion male (in studies that listed these parameters for both survivors and non-survivors) did not change the overall results, although Memis et al. showed a notable adjustment towards a mean difference of zero (Supplementary Fig. S1). Heterogeneity remained high in the adjusted analysis (I^2^ = 91.5%). Visual inspection of funnel plots for the unadjusted and adjusted meta-analyses (Supplementary Figs. S2 and S3) and Egger's regression test (p = 0.66 and p = 0.86 for unadjusted and adjusted plots) did not show any evidence of significant asymmetry although Memis et al.^21^ was noted to be an outlier. A sensitivity analysis with Memis et al. removed still showed a significantly lower serum total cholesterol on day 1 of ICU admission in non-survivors with a pooled mean difference of −17.63 mg/dL (95% CI −24.23 to −11.02) (p < 0.0001, meta-analysis t-distribution), and heterogeneity was reduced but still high (I^2^ = 77.9%) (Supplementary Fig. S4). Meta-regression with Memis et al. included did not show age or proportion male to be significant co-variates (age: beta = 0.80, 95% CI −1.88 to 2.49, p = 0.86, t-distribution) (proportion male: beta = −23.74, 95% CI −160.43 to 112.96, p = 0.73, t-distribution) (Supplementary Figs. S5 and S6), whilst meta-regression without Memis et al. found proportion male (beta = −136.07, 95% CI −238.39 to −33.76, p = 0.01, t-distribution), but not mean age (beta = −0.12, 95% CI −12.0 to 0.95, p = 0.82, t-distribution), to be a significant covariate with mean difference in total cholesterol between non survivors and survivors increasing with proportion of males in studies (Supplementary Figs. S7–S9).

#### HDL-C

**Fig. 3:**
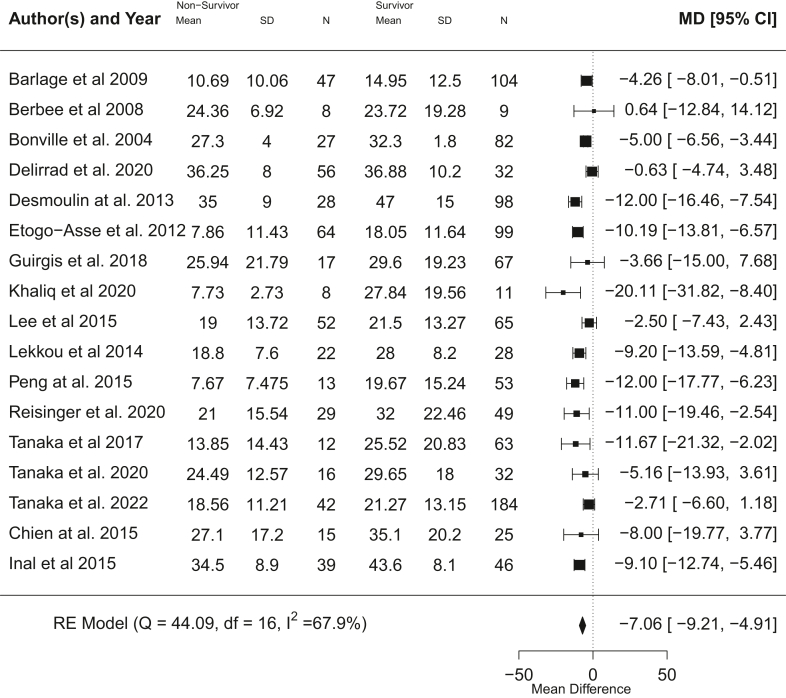
Unadjusted meta-analysis for HDL cholesterol showing pooled mean difference in serum HDL-C and 95% CI between non-survivors and survivors (control) across studies (mg/dL). Heterogeneity shown as I^2^.

Patients that died had a highly significant lower serum HDL-C on day 1 of ICU admission (p < 0.0001, meta-analysis t-distribution) with a pooled mean difference of −7.06 mg/dL (95% CI −9.21 to −4.91 mg/dL) across 17 studies with 1542 participants^17^^,^^20^^,^^22^^,^^31^^,^^32^^,^^34^^,^^36^^,^^37^^,^^40^^,^^41^^,^^43^^,^^45^, ^46^, ^47^, ^48^^,^^50^^,^^51^ (Fig. 3). Moderate heterogeneity was present (I^2^ = 67.9%). Adjustment for proportion male and mean age (in studies that listed these parameters for both survivors and non-survivors) did not show any notable differences in overall meta-analysis trend (Supplementary Fig. S10) and there was moderate heterogeneity (I^2^ = 71.9%). Visual inspection of funnel plots for the unadjusted and adjusted meta-analyses (Supplementary Figs. S11 and S12) and Egger's regression test (p = 0.39 and p = 0.57 for unadjusted and adjusted plots) did not show any evidence of significant publication bias or asymmetry. Meta-regression did not reveal proportion male or mean age as significant co-variates (age: beta = 0.04, 95% CI −0.27 to 0.35, p = 0.79, t-distribution) (proportion male: beta = −21.46, 95% CI −54.20 to 11.28, p = 0.20, t-distribution) (Supplementary Figs. S13 and S14).

#### LDL-C

**Fig. 4:**
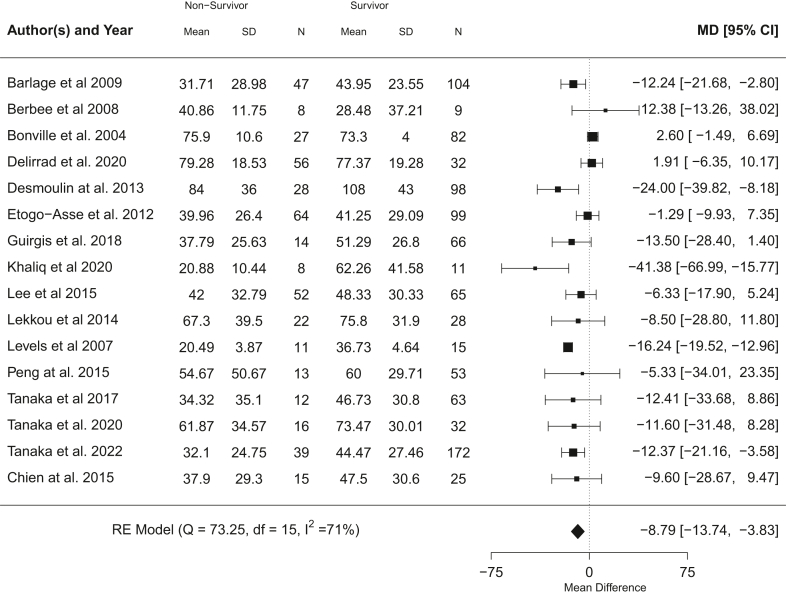
Unadjusted meta-analysis for LDL cholesterol showing pooled mean difference in serum LDL-C and 95% CI between non-survivors and survivors (control) across studies (mg/dL). Heterogeneity shown as I^2^.

Patients that died had significantly lower serum LDL-C on day 1 of ICU admission (p = 0.0005, meta-analysis t-distribution), with a pooled mean difference of −8.79 mg/dL (95% CI, −13.74 to −3.83 mg/dL) across 16 studies with 1386 participants^17^^,^^20^^,^^22^^,^^32^^,^^34^^,^^36^^,^^37^^,^^41^^,^^43^^,^^45^, ^46^, ^47^, ^48^, ^49^, ^50^, ^51^ (Fig. 4). A moderate level of heterogeneity was present (I^2^ = 71%). Adjustment for proportion male and mean age (in studies that listed these parameters for both survivors and non-survivors) did not show any notable differences in overall meta-analysis trend (Supplementary Fig. S15) and heterogeneity was reduced (I^2^ = 44.3%). Visual inspection of funnel plots for the unadjusted and adjusted meta-analyses (Supplementary Figs. S16 and S17) and Egger's regression test (p = 0.36 and p = 0.55 for unadjusted and adjusted plots) did not show any evidence of significant publication bias or asymmetry. Meta-regression found proportion male, but not mean age, as a significant co-variate with mean difference between non-survivor and survivor LDL-C increasing with proportion male in a study (proportion male: beta = −90.83, 95% CI −152.11 to −29.54, p = 0.01, t-distribution) (age: beta = −0.14, 95% CI −0.67 to 0.40, p = 0.61, t-distribution) (Supplementary Figs. S18 and S19).

## Discussion

This systematic review identified 24 studies with 2542 adult patients which were included in pooled quantitative meta-analysis of serum levels of TC, HDL-C or LDL-C cholesterol on the first day of critical care admission. Lower levels of HDL-C (p < 0.0001, meta-analysis t-distribution), LDL-C (p = 0.0005, meta-analysis t-distribution) and total cholesterol (p < 0.0001, meta-analysis t-distribution) on the first day of critical care admission were found to be associated with death. Heterogeneity calculated by the I^2^ statistic was high to moderate, but this is not necessarily detrimental as it demonstrates that across a heterogeneous group of studies and ICU sub-populations, HDL-C and to a lesser extent TC and LDL-C was reliably associated with survival. As a percent change from expected normative levels, HDL-C has an approximately 50% greater lowering of systemic levels in comparison with LDL-C and TC.

Due to data availability, we were only able to adjust our analysis for sex (proportion male) and mean age and found that these did not appear to substantially alter the overall meta-analysis effect trend. There was no evidence of significant publication bias for the analyses of TC, HDL-C and LDL-C, although we acknowledge the limitations of Egger's test for continuous variables in this setting.^55^ Neither male sex, nor age were significant co-variates in the meta-regression for HDL-C. Male sex appeared to be a significant source of heterogeneity for LDL-C and adjusting for age and proportion male reduced heterogeneity substantially (I^2^ 71% to 44.3%). Meta-regression identified male sex as a significant covariate for LDL-C with increased mean difference in LDL-C between non-survivors and survivors as proportion of male subjects increased in studies. For serum total cholesterol, Memis et al.^21^ was identified as a significant outlier. Results were robust to sensitivity analysis with this study excluded, although there was a decrease in the effect size observed (Fig. 2 and Supplementary Fig. S4). Within Memis et al. there was a significant difference between the survivors and non-survivor cohorts at baseline for age (41.7 vs 67.9 years, p < 0.001, unpaired t-test) and sex, with 42% male subjects whereas the other studies all had majority male subjects (range 51–84%) and explains in part the outlier nature of the study. Meta-regression for TC without Memis et al. found male sex to be a significant covariate. Thus, both TC and LDL-C appear to be affected by male sex as a covariate. It is known that pre-menopausal serum LDL-C is lower in women than men and post-menopause LDL-C rises, and may exceed age matched males, likely due to changes in sex hormones.^56^^,^^57^ In contrast, HDL-C does not appear to change significantly with the menopause.^57^^,^^58^ Sex does not appear to have a significant effect on short term mortality in meta-analyses of ICU populations,^59^^,^^60^ however, oestrogen levels are known to be elevated during sepsis and critical illness and correlate with adverse outcomes including mortality.^61^, ^62^, ^63^ We suggest that interactions of oestrogens and other sex hormones on non-HDL lipoproteins during sepsis and critical illness may partially explain the effect of sex as a covariate in our analyses.

The circulatory setpoint levels of TC, HDL-C and LDL-C are governed by the interdependent rates of cellular cholesterol biosynthesis, uptake, efflux, transport, storage, and excretion pathways and where notably HDL-C contributes a rate determinant role in this process. (Note: we use the term *setpoint* rather than baseline and most critically does not refer to how set-point has been incorrectly used to describe predetermined body weight and fat-mass. This is a well-established term for describing the steady state of a dynamical system that is determined by feedforward and feedback processes. It is possible for a system to have multiple setpoints, allowing for adaptation to physiological or environmental changes, and where the trajectory and temporal nature of change may on occasion result in maladaptive changes and under certain conditions can be unstable. Accordingly, setpoint levels more precisely describes the dynamical state and accommodates compensatory changes that can occur by for example hormonal changes or during periods of inflammation or disease). Our results show a coordinate lowering of HDL-C, LDL-C and TC levels associated with poorer outcome and is consistent with a maladaptive regulatory shift. A maladaptive regulatory change unlike dysregulated processes can reset to normative levels. While we have not investigated the restorative changes in the maladaptive-setpoint levels, studies have shown a return to ‘normative’ levels upon recovery from a sepsis episode.^64^ We do not consider other components of measured serum total cholesterol such as remnants and very low-density lipoprotein, and there is a paucity of data on how these components are affected during acute inflammation.^65^

It is worth noting that our results cannot distinguish whether the non-survivors had a pre-existing low setpoint in cholesterol homeostasis and whether this was further exacerbated or acutely occurred at presentation. Population studies and Mendelian Randomisation (MR) work indicate that baseline lower HDL-C but not LDL-C may have a causal association with increased risk and severity of infectious disease,^66^^,^^67^ although interpretation of germline genetic variation associated with HDL-C levels and dynamic changes in HDL during critical illness is not simple. In a recent large scale population study using Nuclear magnetic resonance (NMR) measurements reveals small HDL particle size but not large HDL particle size or LDL particle counts is responsible for the association but is not causal in driving sepsis.^68^ A non-causal role for LDL-C in sepsis is also supported using genetic variants in MR studies.^69^^,^^70^

### A comparison with other systematic reviews

A recent small scale systematic review focusing on HDL-C on admission to ICU, involving 791 participants provides further support for an association of HDL-C with sepsis outcome.^23^ More recently another systematic review of cholesterol levels and sepsis by Hofmaenner et al.,^24^ involving 1283 participants, provides additional agreement with our findings regarding sepsis. The key differences between this review and that of Hofmaenner appears to relate to the slightly more restrictive inclusion criteria we applied and hence the following studies were excluded from our analyses: Yamano et al.^71^ on the basis that this was considered a retrospective investigation, Guirgis et al., 2020^72^ as it was a phase 1 interventional trial of a lipid emulsion, Sharma et al., 2017 and 2019^73^^,^^74^ as patient recruitment and/or lipid sampling in those studies was within 48 hours and not 24 hours of ICU admission and Guirgis et al., 2021^75^ as we lacked patient level data to analyse the subgroup who were admitted to ICU from that study. While our review does not include an analysis of triglyceride levels Hofmaenner et al.^24^ found no association between mortality and admission triglyceride levels. However, in contrast to Hofmaenner et al.^24^ we provide a study quality assessment using the Newcastle Ottawa scale, assess heterogeneity using meta-regression and assessed for evidence of publication bias. Most notably our meta-analysis extends the observed suppression to a much larger sample size (2542 participants) and unlike previous published meta-analysis studies include non-survivors across non-sepsis ICU cohorts, indicating a more general nonspecific host response link of cholesterol homeostasis with inflammation and thus is not exclusive to an infection response. Key differences between this review and Hofmaenner et al. are summarised in Supplementary Table S4.

Thus, sepsis and acute illness in non-survivors leads to a coordinate lowering (around 7–14%) in all major measures of circulatory cholesterol, namely TC, HDL-C and LDL-C. This is consistent with a regulated acute phase inflammatory reaction that can lead to a maladaptive-setpoint of cholesterol metabolism. Key upstream mediators are inflammatory cytokines such as IL-6 and IL-1. Our results of lower levels in non-survivors supports the notion for a greater amplitude and duration of this inflammatory reaction that leads to deaths^76^

### Cholesterol homeostasis, immunometabolism and inflammation

**Fig. 5:**
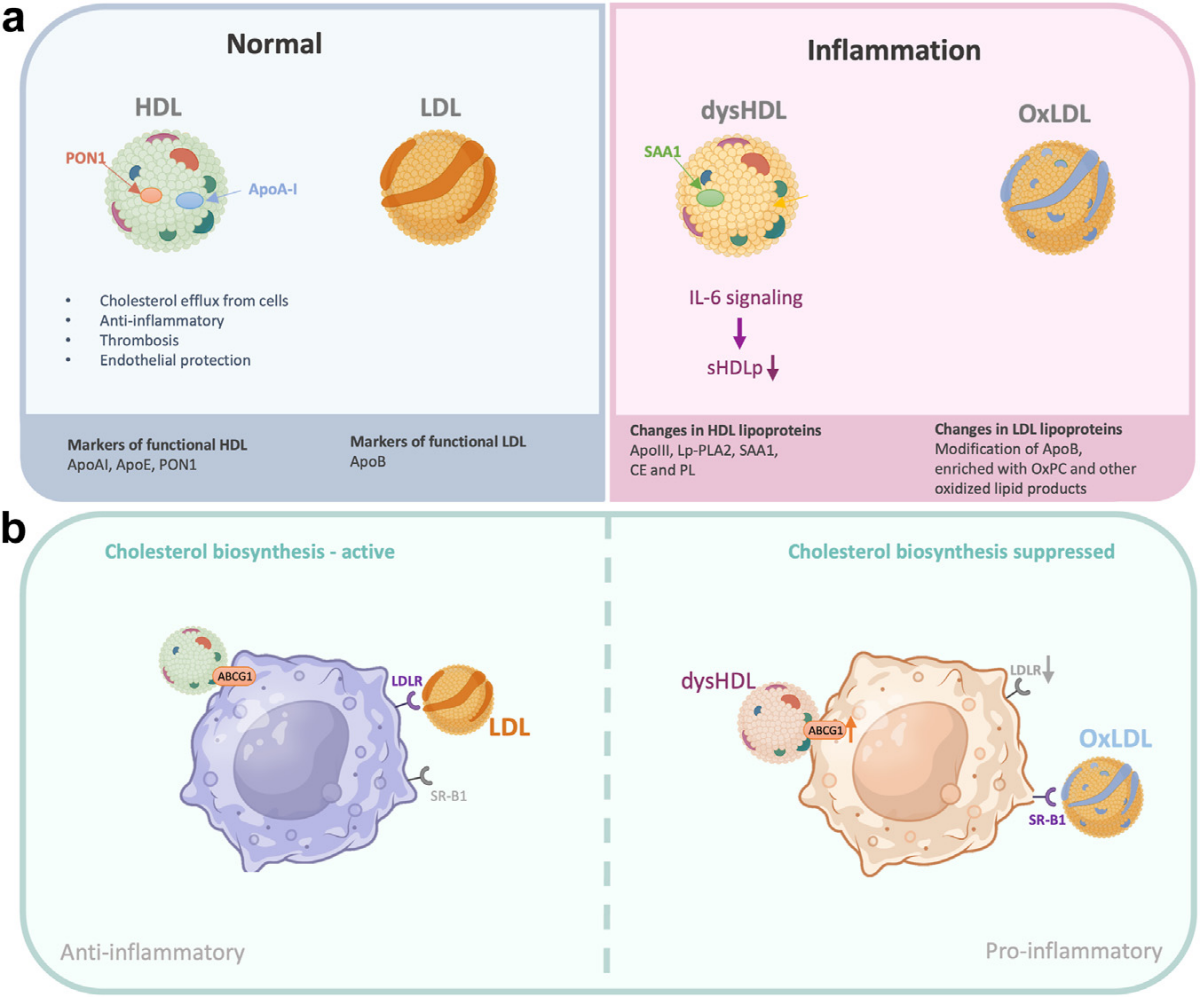
Schematic highlighting normal and inflammatory associated HDL and LDL particle features and macrophage cholesterol immunometabolism. Panel a top left represents normal baseline conditions with healthy cholesterol efflux, anti-inflammatory and cytoprotective functions of HDL particles and associated markers: Apolipoprotein A1 (ApoA1), Apolipoprotein E (ApoE), Paraoxonase 1 (PON1) and LDL with Apolipoprotein B (ApoB). Top right panels illustrates changes that occur in systemic inflammatory states where lower paraoxonase 1 (PON1) levels decrease the capacity of HDL to prevent oxidation of LDL and the exchange of APOA1 with the acute phase Serum Amyloid A (SAA) protein can lead to reduced cholesterol efflux functions; lower levels of cholesterol esters (CE) and phospholipids (PL); number of small HDL particles (sHDLp) and potential development of ‘pro-inflammatory’ subclass of HDL (dysHDL) with associated marker of SAA and increased proportion of oxidised LDL (oxLDL) and phosphotidylcholine (oxPC). Arrows indicate causal drivers of IL-6 signalling in reducing small HDL particle numbers. ApoIII = Apolipoprotein C-III, SAA1 = serum amyloid A1, Lp-PLA2 = Lipoprotein-Associated Phospholipase A2. Panel b illustrates the immunometabolism of cholesterol biosynthesis and the delivery of cholesterol via LDL binding its cognate receptor (LDLR) and in inflammatory states, where LDLR is downregulated, oxLDL has preferential uptake via the Scavenger Receptor (SR-B1). The efflux of cholesterol to HDL in macrophage uses primarily the cholesterol exporter (ABCG1) that in inflammatory conditions is upregulated in conjunction with an increased proportion of dysfunctional HDL particles (dysHDLp) that have lost their anti-inflammatory features. The linkage to immunometabolism is indicated by the change in flux of cholesterol biosynthesis and its association with macrophage immunophenotype switching between pro and anti-inflammatory states.

Structural and functional changes of HDL and LDL occur during inflammation and acute phase response (Fig. 5a). During inflammation the most prominent changes take place for HDL and associated lipids,^77^^,^^78^ although changes in LDL can also occur and include increased oxidation of LDL to a more pro-inflammatory phenotype and an increase in levels of the pro-inflammatory Lipoprotein (a).^77^, ^78^, ^79^, ^80^ The measurements of HDL-C and LDL-C in our analyses does not account for these morphological or functional changes. While the biological role for these changes is not precisely known it is clear they are under inflammatory control, where IL-6 signalling has been shown recently to explain the reduction in small HDL particle numbers.^68^

Cellular cholesterol biosynthesis has a central rate determining role in cholesterol homeostasis, and flux activity and products of the pathway are integral to host-protection governing inflammatory and adaptive immune cell functions.^4^^,^^5^ Notably, the cross regulation between inflammation and cholesterol metabolism in macrophages and other immune cells (termed immunometabolism) is well established and where in acute infection Toll Like Receptor and interferon signalling coordinately suppresses flux in cholesterol biosynthesis and stimulates the production of oxysterols that directly control innate and adaptive immune reactions, including the switching of adaptive immune cell functions from inflammatory (IFN gamma) to resolving (IL-10) phenotypes.^4^^,^^5^ In this regard small HDL particles have a vital contribution in controlling the efflux of cholesterol^81^ and hence is tightly linked to feedback regulation of flux in cholesterol biosynthesis in immune cells (Fig. 5b). Furthermore, oxidised LDL can further promote pro-inflammatory effects (Fig. 5b). In particular, flux control in macrophage cholesterol biosynthesis is central to determining the immunophenotype of macrophages and activation of the inflammatory cascade.^4^^,^^5^ Accordingly, from an immunometabolism perspective prolonged hypocholesteremia has the risk of potentially driving macrophage states from a pro-inflammatory to anti-inflammatory phenotype. Understanding how systemic changes are mechanistically interlinked with immunometabolism of innate and adaptive cellular effector functions is incomplete and is an area that warrants further research.

### Prognostic metabolic biomarkers in sepsis and critical illness—a potential role for HDL associated biomarkers

Nuclear magnetic resonance (NMR) measurement of HDL particle count and particle size, indicate that pre-existing low number of small HDL particles increases the risk of death from infectious disease.^68^^,^^82^ However, while small HDL particles are the most relevant form in terms of function in a very recent study, enumeration of HDL particle size by NMR, has shown not to have any causal association with death from sepsis^68^ and may point to a “red herring” for diagnostics and therapeutic intervention. However, the efficacy of HDL particles is strongly dependent on the local environment partially driven by Apolipoprotein A1 (ApoA1), Paraoxonase 1 (PON1), and lecithin-cholesterol acyltransferase (LCAT) activity.^83^^,^^84^ Therefore, the functionality of HDL particles is important in addition to HDL quantity or size. The accurate measurement of HDL particle size is not trivial and instrumentation such as NMR present significant challenges (both technical and financial) for a near term diagnostic platform. However, this does not rule out a role for other HDL associated biomarkers. In this connection, studies of the HDL lipidome have revealed that cholesteryl esters (30–40% of total lipid), rather than free cholesterol (5–10%) predominate and account for the major proportion of cholesterol in the small HDL particles^84^ and could potentially offer a more readily implementable HDL-biomarker. Circulatory levels of esterified cholesterol are also an excellent functional marker of LCAT enzyme activity that is responsible for producing lysophospholipids on small HDL particles.^84^ Inflammatory induced phospholipases are associated with reduced HDL cholesterol and are associated with changes in lysophospholipid composition which are further imbued with pro- and anti-inflammatory activity.^84^ A more complete characterisation of the HDL lipidome in chronic and acute inflammation is warranted. As a cautionary note, HDL-cholesterol levels and to a lesser extent NMR measured HDL are not completely accurate indicators of the concentrations of various functional and physical subtypes of HDL.^85^ Nevertheless, comparisons within a given methodology consistently reveals an association of low HDL with death from sepsis and critical illness. Therefore, future clinical studies investigating the prognostic utility of HDL-based biomarkers, in combination with other host-based biomarkers, is strongly warranted.

### Therapeutic potential of HDL

Evidence for a direct causal role of HDL-C in contributing toward a maladaptive host response is more controversial. As mentioned above a recent large population study of 270,000 participants with NMR based data showed that low levels of small HDL particles have no causal role with sepsis and sepsis deaths.^68^ Critically, this study reveals that low levels of small HDL particles are causally linked to IL-6 signalling (Fig. 5). As a corollary, in an acute infection a large IL-6 driven inflammatory response would lead to a strong suppression of HDL and is suggestive that low levels in sepsis is a bystander effect of high inflammatory reaction. A possible protective role of HDL-C in sepsis cannot be ruled out. In this connection clinical genetics and murine models that demonstrate that inhibition of the cholesteryl ester transfer protein (CETP), whose function is associated with decreases in cholesterol esters and circulating HDL-C, is protective against mortality in acute sepsis.^86^ In contrast gain-of-function mutations affecting CETP, leading to decreased HDL-C, are associated with increased risk of mortality due to sepsis.^86^ Gaining more precise mechanistic insight into the multi-faceted relationship between sepsis and HDL and host immunometabolism and whether this represents a protective inflammatory response or pathological change may identify novel therapeutic targets and interventions for use in critical illness.

The notion of increasing circulatory levels of HDL in patients with sepsis was first piloted in preclinical models using reconstituted HDL^87^ but failed clinical translation due to endotoxin contamination, low purity and pharmacokinetic properties. More recently, the increasing number of clinical and pre-clinical studies involving the administration of synthetic HDL particles, that show high clinical grade purity and safety, in acute coronary syndrome has rekindled interests in sepsis.^11^^,^^26^^,^^88^, ^89^, ^90^, ^91^, ^92^ These studies showed not only an attenuating effect of synthetic HDL on endotoxin shock but also the suppression of inflammatory signalling in macrophages and endothelial cells, raising potential for a clinical translatable therapy. However, we propose that it would be prudent before embarking on therapeutic trials for sepsis, to precisely delineate if causality is associated with specific HDL functional class(es) and risk of mortality. As caveat emptor, trial failure risks curtailing key research to uncover critical mechanistic links.

### Limitations

A significant limitation relates to the inability to fully control for patient characteristics that could simultaneously affect both outcome variable, mortality, and the serum concentrations of HDL-C, LDL-C, and total cholesterol. Whilst we are aware of, and actively sought evidence for the effects of potential confounders whilst assessing study quality, we could not assess this at an individual patient level and adjust for factors other than age and sex. Similarly we were only able to utilise mean age and male sex as covariables in our meta-regression. There was also variability amongst the 24 studies synthesised for meta-analysis in accounting for the use of cholesterol lowering drugs such as statins. Of these only seven excluded patients using statins or excluded hyperlipidemia as a comorbidity and had no statin use.^17^^,^^32^^,^^40^^,^^41^^,^^43^^,^^44^^,^^46^ Four additional studies^22^^,^^31^^,^^48^^,^^50^ reported on statin use in their cohort, of which two of them^22^^,^^31^ assessed whether there was a relationship between statin use and survival and concluded there was no significant difference.

### Conclusions and future research

We provide a strong basis to support that a drop in HDL-C levels either prior to or early during sepsis and critical illness has prognostic value. We suggest that subclass specific HDL-biomarkers could have clinical utility when used alongside existing metabolic markers such as lactate in the early detection of sepsis and development of septic shock. We propose future investigations should consider circulatory levels of cholesterol esters as a possible readily implementable HDL associated biomarker. Future work should also aim to precisely define HDL functional subclasses and associated biomarkers that could be used as a reference range below which serum HDL cholesterol is significantly suppressed and likely to be associated with severe illness. In this regard, with increasing interest in exploring synthetic HDL and lipid supplementation as therapeutic interventions for sepsis, consideration of the available evidence for both association and a causation in sepsis is called for. Hence, in addition to quantitative lipoprotein analyses, functional assays for HDL particles should also be investigated. Our analysis was restricted to adults, and although limited studies in neonates can be found,^93^ the question remains for future studies of whether similar observations are evident in early life. We also highlight areas in which further research can be more rigorous, through careful selection of patient cohorts and adjustment for relevant co-morbidities which may affect both lipid profile and ICU outcome and measuring more precisely the various functional and structural subclasses of HDL.

## Contributors

RT, CZ, DG, MA, SK, were involved in the methodology of the work, data curation, analysis and interpretation. PR for graphical support and ACR for unpublished data. DW and FH were involved in the conceptualisation, editing and interpretation. TF and JW contributed to the methodology of the work and to statistical data analysis and interpretation. PG and DG were involved in the conceptualisation and methodology of the work, data analysis and interpretation. All authors contributed to the writing and review of the final manuscript.

RT, SK, JW and PG had direct access to and verified the underlying data reported in the manuscript.

## Data sharing statement

All relevant data are included in the manuscript and Supplementary files.

## Declaration of interests

Peter Ghazal is a member of the development board for Sepsis Trust UK with no cash incentive, Received funding from the EDRF Ser Cymru (Welsh Government) programme, EU-FP7 projects: ClouDx-I and NeoVanc. Thorsten Förster is an inventor of patent US20170022568—“Molecular Predictors Of Sepsis” and is declared as an existing interest in intellectual property around transcriptomic markers of sepsis, but is not related to the current work beyond the broad theme of sepsis.

## Appendix ASupplementary data

Supplementary Data and Tables S1–S4

Supplementary Figs. S1–S19 **Supplementary Fig. 1**: adjusted meta-analysis for total cholesterol showing mean difference in serum total cholesterol between non-survivors and survivors (control) across studies (mg/dL) adjusted for mean age and proportion male subjects for studies which had this data available in the manuscript. Heterogeneity shown as I^2^. **Supplementary Fig. 2**: funnel plot for unadjusted Total Cholesterol meta-analysis showing study standard error plotted against mean difference between survivor and non-survivor groups for day 1 ICU total cholesterol. **Supplementary Fig. 3**: funnel plot for adjusted Total Cholesterol meta-analysis showing study standard error plotted against mean difference between survivor and non-survivor groups for day 1 ICU total cholesterol. **Supplementary Fig. 4**: unadjusted meta-analysis for total cholesterol, with Memis et al excluded, showing pooled mean difference in serum total cholesterol and 95% CI between non-survivors and survivors (control) across studies (mg/dL). Heterogeneity shown as I^2^. **Supplementary Fig. 5:** meta-regression for total cholesterol showing effect of mean age (years) as a covariable. Shaded area indicates the 95% confidence band. **Supplementary Fig. 6**: meta-regression for total cholesterol, with showing effect of sex (proportion male) as a covariable. Shaded area indicates the 95% confidence band. **Supplementary Fig. 7**: adjusted meta-analysis for total cholesterol, without Memis et al, showing mean difference in serum total cholesterol between non-survivors and survivors (control) across studies (mg/dL) adjusted for mean age and proportion male subjects for studies which had this data available in the manuscript. Heterogeneity shown as I^2^. **Supplementary Fig. 8**: meta-regression for total cholesterol, without Memis et al, showing effect of mean age (years) as a covariable. Shaded area indicates the 95% confidence band. **Supplementary Fig. 9**: meta-regression for total cholesterol, without Memis et al, showing effect of sex (proportion male) as a covariable. Shaded area indicates the 95% confidence band. **Supplementary Fig. 10:** adjusted meta-analysis for HDL-C showing mean difference in serum HDL-C between non-survivors and survivors (control) across studies (mg/dL) adjusted for mean age and proportion male subjects for studies which had this data available in the manuscript. Heterogeneity shown as I^2^. **Supplementary Fig. 11**: funnel plot for unadjusted HDL-C meta-analysis showing study standard error plotted against mean difference between survivor and non-survivor groups for day 1 ICU HDL-C. **Supplementary Fig. 12**: funnel plot for adjusted HDL-C meta-analysis showing study standard error plotted against mean difference between survivor and non-survivor groups for day 1 ICU HDL-C. **Supplementary Fig. 13**: meta-regression for HDL-C showing effect of mean age (years) as a covariable. Shaded area indicates the 95% confidence band. **Supplementary Fig. 14**: meta-regression for HDL-C, with showing effect of sex (proportion male) as a covariable. Shaded area indicates the 95% confidence band. **Supplementary Fig. 15**: adjusted meta-analysis for LDL-C showing mean difference in serum LDL-C between non-survivors and survivors (control) across studies (mg/dL) adjusted for mean age and proportion male subjects for studies which had this data available in the manuscript. Heterogeneity shown as I^2^. **Supplementary Fig. 16**: funnel plot for unadjusted LDL-C meta-analysis showing study standard error plotted against mean difference between survivor and non-survivor groups for day 1 ICU LDL-C. **Supplementary Fig. 17**: funnel plot for adjusted LDL-C meta-analysis showing study standard error plotted against mean difference between survivor and non-survivor groups for day 1 ICU LDL-C. **Supplementary Fig. 18**: meta-regression for LDL-C, with showing effect of sex (proportion male) as a covariable. Shaded area indicates the 95% confidence band. **Supplementary Fig. 19**: meta-regression for LDL-C showing effect of mean age (years) as a covariable. Shaded area indicates the 95% confidence band.
