# Supplementary material for: Low circulatory levels of total cholesterol, HDL-C and LDL-C are associated with death of patients with sepsis and critical illness: systematic review, meta-analysis, and perspective of observational studies
Source: eBioMedicine. 2024 Jan 29;100:104981. doi: 10.1016/j.ebiom.2024.104981 (PMC10844818; doi:10.1016/j.ebiom.2024.104981)
Supplement: Supplementary Data and Tables S1–S4 [file mmc1.docx]

**Supplementary Material**

**Low circulatory levels of total cholesterol, HDL-C and LDL-C are associated with death of patients with sepsis and critical illness: Systematic review, meta-analysis, and perspective of observational studies.**

**Rory Taylor*^1^**† **, Chengyuan Zhang^2^**†**, Deslit George^3^**†**, Sarah Kotecha^4^, Mariam Abdelghaffar^5^, Thorsten Forster^6^, Patricia Dos Santos Rodrigues^3^, Alexander C. Reisinger^7^, Daniel White^8^, Fergus Hamilton^9^, W. John Watkins^10^, David M Griffith^11^, Peter Ghazal*^8^**

^1^ Deanery of Biomedical Sciences, University of Edinburgh Medical School, Edinburgh, UK

^2^ Department of Anaesthesia, Critical Care and Pain Medicine, NHS Lothian, Edinburgh, UK

^3^ School of Medicine, University of Cardiff, Cardiff, United Kingdom.

^4^ Department of Child Health, School of Medicine, University of Cardiff, Cardiff, United Kingdom.

^5^ School of Medicine, Royal College of Surgeons in Ireland, Bahrain

^6^ LifeArc, Edinburgh Bioquarter, Edinburgh, UK

^7^ Department of Internal Medicine, Intensive Care Unit, Medical University of Graz, Graz, Austria

^8^ Project Sepsis, Systems Immunity Research Institute, School of Medicine, University of Cardiff, Cardiff, UK

^9^ MRC Integrative Epidemiology Unit, University of Bristol, Bristol, UK

^10^ Dept of Immunity and Infection, School of Medicine, Cardiff University, Cardiff, UK

^11^ Anaesthesia, Critical Care and Pain, Molecular, Genetics, and Population Health Sciences, University of Edinburgh, Edinburgh, UK

*** Correspondence:**Correspondence to: Peter Ghazal. Email: GhazalP@cardiff.ac.uk (ORCID 0000-0003-0035-2228)

# Supplementary Data

**Search strategy logic**

((Cholesterol or HDL or LDL or Lipoprotein or Lipoproteins) AND (Intensive Care or Intensive Care Units or Critical Care or Critical Care or ITU or ICU or Critical Illness or Critically Ill or Sepsis or Septi*)).ti,ab

This was adapted slightly for each database.

## Supplementary Tables

**Supplementary Table 1: MOOSE checklist for reporting Meta-analyses of Observational Studies.**

Note page number refers to the submitted manuscript and may differ from online content.

| **Item No** | **Recommendation** | | **Reported on Page No** | |
| --- | --- | --- | --- | --- |
| Reporting of background should include | | | | |
| 1 | Problem definition | | 3-4 | |
| 2 | Hypothesis statement | | - | |
| 3 | Description of study outcome(s) | | 4-5 | |
| 4 | Type of exposure or intervention used | | 5 | |
| 5 | Type of study designs used | | 5 | |
| 6 | Study population | | 5 | |
| Reporting of search strategy should include | | | | |
| 7 | Qualifications of searchers (eg, librarians and investigators) | | 1 | |
| 8 | Search strategy, including time period included in the synthesis and key words | | 4 | |
| 9 | Effort to include all available studies, including contact with authors | | 4 | |
| 10 | Databases and registries searched | | 4 | |
| 11 | Search software used, name and version, including special features used (eg, explosion) | | 4, supplementary data | |
| 12 | Use of hand searching (eg, reference lists of obtained articles) | | 4 | |
| 13 | List of citations located and those excluded, including justification | | Table 1, Supplementary Table 3 | |
| 14 | Method of addressing articles published in languages other than English | | 5 | |
| 15 | Method of handling abstracts and unpublished studies | | 4-5 | |
| 16 | Description of any contact with authors | | 4,6 | |
| Reporting of methods should include | | | | |
| 17 | Description of relevance or appropriateness of studies assembled for assessing the hypothesis to be tested | | 4-5 | |
| 18 | Rationale for the selection and coding of data (eg, sound clinical principles or convenience) | | 5 | |
| 19 | Documentation of how data were classified and coded (eg, multiple raters, blinding and interrater reliability) | | - | |
| 20 | Assessment of confounding (eg, comparability of cases and controls in studies where appropriate) | | 5 | |
| 21 | Assessment of study quality, including blinding of quality assessors, stratification or regression on possible predictors of study results | | 5 | |
| 22 | Assessment of heterogeneity | | 5-6 | |
| 23 | Description of statistical methods (eg, complete description of fixed or random effects models, justification of whether the chosen models account for predictors of study results, dose-response models, or cumulative meta-analysis) in sufficient detail to be replicated | | 5-6 | |
| 24 | Provision of appropriate tables and graphics | | Figure 1 | |
| Reporting of results should include | | | | |
| 25 | Graphic summarizing individual study estimates and overall estimate | | Figs 2-4 | |
| 26 | Table giving descriptive information for each study included | | Table 1 | |
| 27 | Results of sensitivity testing (eg, subgroup analysis) | | Supplementary Figures 4, 7, 8, 9 | |
| 28 | Indication of statistical uncertainty of findings | | 7-8 | |
| Reporting of discussion should include | | | | |
| 29 | | Quantitative assessment of bias (eg, publication bias) | | 9 |
| 30 | | Justification for exclusion (eg, exclusion of non-English language citations) | | 10, Supplementary Table 3 |
| 31 | | Assessment of quality of included studies | | 7, 12 |
| Reporting of conclusions should include | | | | |
| 32 | | Consideration of alternative explanations for observed results | | 9, 10, 12 |
| 33 | | Generalization of the conclusions (ie, appropriate for the data presented and within the domain of the literature review) | | 13 |
| 34 | | Guidelines for future research | | 12, 13 |
| 35 | | Disclosure of funding source | | 6 |

*From*: Stroup DF, Berlin JA, Morton SC, et al, for the Meta-analysis Of Observational Studies in Epidemiology (MOOSE) Group. Meta-analysis of Observational Studies in Epidemiology. A Proposal for Reporting. *JAMA*. 2000;283(15):2008-2012. doi: 10.1001/jama.283.15.2008.

**Supplementary Table 2: Newcastle Ottawa Scale adaptation and rating**

results of assessment of quality of included studies using a bespoke version of the Newcastle-Ottawa Scale. Each item for “Selection” can receive a maximum of one star. There are two stars available for “Comparability”. Each item for “outcome” can receive a maximum of one star. The maximum score for a study is 9 stars.

**Selection**

**1) Representativeness of the exposed cohort**

a) truly representative of a general ICU cohort (mixed medical/surgical ICU cohort)*

b) somewhat representative of a general ICU cohort (mixed medical/surgical cohort)*

c) not representative of the average patient admitted to ICU e.g. selected from normal hospital population, community

d) no description of the derivation of the cohort

**2) Selection of the non exposed cohort**

a) drawn from the same population as the exposed cohort *

b) drawn from a different population as the exposed cohort*

c) no description of the derivation of the non-exposed cohort

**3) Ascertainment of exposure (serum HDL quantification)**

a) samples drawn prospectively and then assessed in clinical laboratory*

b) values collected retrospectively from patient notes/records*

**4) Demonstration that outcome of interest (life threatening critical illness) was not present at start of study**

a) yes *

b) no

**Comparability**

**1) Comparability of cohorts on the basis of the design or analysis**

a) study controls for key confounding factors known to influence lipid profile and ICU prognosis* (chronic renal disease, chronic liver disease, age)

b) study controls for any additional confounding factor * (coronary artery disease, statin/other lipid modulating therapy used within past 7 days, history of dyslipidaemia, malignancy)

**Outcome**

**1) Assessment of outcome (mortality)**

a) independent blind assessment*

b) record linkage*

c) within specified time-frame (i.e. 28 day, until hospital discharge) *

d) no description

**2) Was follow-up long enough for outcome (mortality) to occur**

a) yes (at least 14 days or until discharge) *

b) no

**3) Adequacy of follow up of participants**

a) complete follow up - all subjects accounted for*

b) subjects lost to follow up unlikely to introduce bias - small number lost <5%*

c) 5-20% lost but good description of those lost and unlikely to introduce bias*

d) follow up rate <80%

e) no statement

**Justification for tool.**

The above tool is based upon the standard NOS template. We discuss or justify modifications outwith the standard recommendations below.

**Comparability**: this is split into two categories: key confounding factors and additional confounding factors. Key confounding factors were those with recognised evidence that they influence both lipid profiles and ICU outcome – chronic renal disease (1-3) and chronic liver disease (4-7) and age (8-12). Additional confounding factors are those that do not fill the criteria for key confounding factors but could have a role as confounding. Dyslipidaemia and lipid modulating therapies are included for their direct effects or associations with altered lipid profiles. Coronary artery disease also has a recognised association with lipid profiles although the effect on ICU prognosis is less clear. Certain malignancies are associated with altered lipid profiles and/or ICU prognosis but given the heterogenous natures of various malignant illnesses we include Malignancy under additional rather than key confounding factors.

**Assessment of Outcome**: we include option (C) which is not under the base NOS template. We did this for two reasons, firstly given that cholesterol levels were assessed by standardised clinical laboratory equipment we did not think assessment blinding was vital to study quality. Secondly given that mortality of hospital inpatients is not an outcome where there is significant uncertainty we allowed studies which assessed in-hospital mortality or short term mortality (within 30 days) to receive a star in this category

|  | **Selection** | | | | **Comparability** | **Outcome** | | |  |
| --- | --- | --- | --- | --- | --- | --- | --- | --- | --- |
| Author | Representativeness of the exposed cohort | Selection of the non exposed cohort | Ascertainment of exposure | Demonstration that outcome of interest was not present at the start of the study | Comparability of cohorts on the basis of design or analysis | Assessment of outcome | Was follow-up long enough for outcomes to occur | Adequacy of follow up of cohorts | No. of stars |
| Barati et al. 2013 | ***** | ***** | ***** |  |  |  | ***** | ***** | 5 |
| Barlage et al. 2009 | ***** | ***** | ***** |  |  | ***** | ***** | ***** | 6 |
| Berbee et al. 2008 | ***** | ***** | ***** |  |  |  | ***** | ***** | 5 |
| Biller et al. 2014 | ***** | ***** | ***** |  |  | ***** | ***** | ***** | 6 |
| Bonville et al. 2004 | ***** | ***** | ***** |  |  |  | ***** | ***** | 5 |
| Chien et al. 2005 | ***** | ***** | ***** |  | ***** | ***** | ***** | ***** | 7 |
| Chien et al. 2015 | ***** | ***** | ***** |  | ***** | ***** | ***** | ***** | 7 |
| Delirrad et al. 2020 | ***** | ***** | ***** |  |  |  | ***** | ***** | 5 |
| Desmoulin et al. 2013 |  | ***** | ***** |  |  | ***** | ***** | ***** | 5 |
| Etogo-Asse et al. 2012 | ***** | ***** | ***** |  |  |  | ***** | ***** | 5 |
| Fraunberger et al. 2000 | ***** | ***** | ***** |  |  | ***** | ***** | ***** | 6 |
| Gordon et al. 2001 | ***** | ***** | ***** |  |  |  | ***** | ***** | 5 |
| Guimaraes et al. 2008 | ***** | ***** | ***** |  | ***** | ***** | ***** | ***** | 7 |
| Guirgis et al. 2018 | ***** | ***** | ***** |  |  | ***** | ***** | ***** | 6 |
| Inal et al. 2015 | ***** | ***** | ***** |  |  | ***** | ***** | ***** | 6 |
| Khaliq et al. 2020 | ***** | ***** | ***** |  |  | ***** | ***** | ***** | 6 |
| Lee et al. 2015 | ***** | ***** | ***** |  |  | ***** | ***** | ***** | 6 |
| Levels et al. 2007 | ***** | ***** | ***** |  |  | ***** | ***** | ***** | 6 |
| Lekkou et al. 2014 | ***** | ***** | ***** |  |  | ***** | ***** | ***** | 6 |
| Luthold et al. 2007 | ***** | ***** | ***** |  |  |  | ***** | ***** | 5 |
| Memis et al. 2007 | ***** | ***** | ***** |  |  |  | ***** | ***** | 5 |
| Oliva et al 2022 | ***** | ***** | ***** |  |  | ***** | ***** | ***** | 6 |
| Peng et al. 2015 | ***** | ***** | ***** |  |  | ***** | ***** | ***** | 6 |
| Reisinger et al. 2020 | ***** | ***** | ***** |  |  | ***** | ***** | ***** | 6 |
| Tachyla et al. 2017 | ***** | ***** | ***** |  |  |  | ***** | ***** | 5 |
| Tanaka et al. 2017 | ***** | ***** | ***** |  |  |  | ***** | ***** | 5 |
| Tanaka et al. 2020 | ***** | ***** | ***** |  |  |  | ***** | ***** | 5 |
| Tanaka et al. 2022 | ***** | ***** | ***** |  |  | ***** | ***** | ***** | 6 |
| Uzundere et al. 2015 | ***** | ***** | ***** |  |  | ***** | ***** | ***** | 6 |

**References (Supplementary Table 2):**

1. Clermont G, Acker CG, Angus DC, et al. Renal failure in the ICU: Comparison of the impact of acute renal failure and end-stage renal disease on ICU outcomes. *Kidney Int*. 2002 Sep;62(3):986–96.

2. Arulkumaran N, Annear NMP, Singer M. Patients with end-stage renal disease admitted to the intensive care unit: systematic review. *Br J Anaesth*. 2013 Jan 1;110(1):13–20.

3. Strijack B, Mojica J, Sood M, et al. Outcomes of chronic dialysis patients admitted to the intensive care unit. *Journal of the American Society of Nephrology.* 2009 Nov;20(11):2441–7.

4. Ghadir MR, Riahin AA, Havaspour A, et al. The relationship between lipid profile and severity of liver damage in cirrhotic patients. *Hepatitis monthly.* 2010;10(4):285–8.

5. Chrostek L, Supronowicz L, Panasiuk A, et al. The effect of the severity of liver cirrhosis on the level of lipids and lipoproteins. *Clin Exp Med*. 2014 Nov;14(4):417–21.

6. O’Brien AJ, Welch CA, Singer M, et al. Prevalence and outcome of cirrhosis patients admitted to UK intensive care: a comparison against dialysis-dependent chronic renal failure patients. *Intensive Care Med*. 2012 Jun 29;38(6):991–1000.

7. Shellman RG, Fulkerson WJ, DeLong E, et al. Prognosis of patients with cirrhosis and chronic liver disease admitted to the medical intensive care unit. *Crit Care Med*. 1988 Jul;16(7):671–8.

8. Peigne V, Somme D, Guérot E, et al. Treatment intensity, age and outcome in medical ICU patients: results of a French administrative database. *Annals of intensive care*. 2016 Dec;6(1):7.

9. Fuchs L, Chronaki CE, Park S, et al. ICU admission characteristics and mortality rates among elderly and very elderly patients. *Intensive Care Med*. 2012 Oct;38(10):1654–61.

10. Ferrara A, Barrett-Connor E, Shan J. Total, LDL, and HDL cholesterol decrease with age in older men and women. The Rancho Bernardo Study 1984-1994. *Circulation*. 1997 Jul 1;96(1):37–43.

11. Weijenberg MP, Feskens EJ, Kromhout D. Age-related changes in total and high-density-lipoprotein cholesterol in elderly Dutch men. *American journal of public health*. 1996 Jun;86(6):798–803.

12. Upmeier E, Lavonius S, Heinonen P, et al. Longitudinal changes in serum lipids in older people The Turku Elderly Study 1991–2006. Age and ageing. 2011. 40(2):280–3.

**Supplementary Table 3: Excluded studies**

full text excluded studies listed by first author, with reason for exclusion, see supplementary data files for reference list.

| **Study** | **Reason for exclusion** |
| --- | --- |
| Begue et al 2023 (1) | No assessment of cholesterol levels and mortality |
| Chenaud et al 2004 (2) | No assessment of cholesterol levels and mortality |
| Drobnik et al 2003 (3) | Lipids not assessed <24hrs ICU admission |
| Gong et al 2019 (4) | Non ICU cohort |
| Guirgis et al 2021 (5) | Mortality assessment not at required timepoints |
| Hazrati et al 2018 (6) | Mortality assessment not at required timepoints |
| Li et al 2022 (7) | Non ICU cohort |
| Montero-Chacon et al 2020 (8) | Retrospective cohort |
| Mousa et al 2022 (9) | Data discrepancies in paper |
| Novak et al 2010 (10) | Lipids not assessed <24hrs ICU admission |
| Prado et al 2023 (11) | No assessment of cholesterol and mortality |
| Reisinger et al 2021 (12) | Same dataset as another included study |
| Samara et al 2010 (13) | No assessment of cholesterol levels and mortality |
| Schrutka et al 2016 (14) | No assessment of cholesterol levels and mortality |
| Sharma et al 2017 (15) | Lipids not assessed <24hrs ICU admission |
| Sharma et al 2019 (16) | Lipids not assessed <24hrs ICU admission |
| Tanaka et al 2021 (17) | Lipids not assessed <24hrs ICU admission |
| Tsai et al 2009 (18) | Mortality assessment not at required timepoints |
| Yamano et al 2016 (19) | Retrospective cohort |

**References for Supplementary Table 3**

1.      Begue F, Chemello K, Veeren B, Lortat-Jacob B, Tran-Dinh A, Zappella N, et al. Plasma Apolipoprotein Concentrations Are Highly Altered in Severe Intensive Care Unit COVID-19 Patients: Preliminary Results from the LIPICOR Cohort Study. Int J Mol Sci. 2023;24(5):4605.

2.      Chenaud C, Merlani PG, Roux-Lombard P, Burger D, Harbarth S, Luyasu S, et al. Low apolipoprotein A-I level at intensive care unit admission and systemic inflammatory response syndrome exacerbation. Crit Care Med. 2004;32(3):632–7.

3.      Drobnik W, Liebisch G, Audebert FX, Fröhlich D, Glück T, Vogel P, et al. Plasma ceramide and lysophosphatidylcholine inversely correlate with mortality in sepsis patients. J Lipid Res. 2003;44(4):754–61.

4.      Gong Y, Ding F, Gu Y. Can Serum Nutritional Related Biomarkers Predict Mortality Of Critically Ill Older Patients With Acute Kidney Injury? Clin Interv Aging. 2019;14:1763–9.

5.      Guirgis FW, Dodani S, Leeuwenburgh C, Moldawer L, Bowman J, Kalynych C, et al. HDL inflammatory index correlates with and predicts severity of organ failure in patients with sepsis and septic shock. PLoS One. 2018 Sep 1;13(9).

6.      Hazrati E, Noorifard M, Afsahi M, Rohi A, Rafiei M. The relationship between lipid profile and CRP in severe sepsis with patient prognosis. Journal of Advanced Pharmacy Education and Research. 2018;8(1):103–7.

7.      Li Z, Luo Z, Shi X, Pang B, Ma Y, Jin J. The Levels of Oxidized Phospholipids in High-Density Lipoprotein During the Course of Sepsis and Their Prognostic Value. Front Immunol. 2022;13:893929.

8.      Montero-Chacon LB, Padilla-Cuadra JI, Chiou SH, Torrealba-Acosta G. High-Density Lipoprotein, Mean Platelet Volume, and Uric Acid as Biomarkers for Outcomes in Patients With Sepsis: An Observational Study. J Intensive Care Med. 2020;35(7):636–42.

9.    Mousa SAE hamid, Hassan NA, Abd El-Hafez A, Gouda TES. Relation Between Nutritional Status and Outcome in Critically ill Patient. NeuroQuantology. 2022 Nov;20(15).

10.    Novak F, Vavrova L, Kodydkova J, Novak FS, Hynkova M, Zak A, et al. Decreased paraoxonase activity in critically ill patients with sepsis. Clin Exp Med. 2010;10(1):21–5.

11.    Prado Y, Tapia P, Eltit F, Reyes-Martinez C, Feijoo CG, Llancalahuen FM, et al. Sepsis-Induced Coagulopathy Phenotype Induced by Oxidized High-Density Lipoprotein Associated with Increased Mortality in Septic-Shock Patients. Antioxidants (Basel). 2023;12(3).

12.    Reisinger AC, Schuller M, Sourij H, Stadler JT, Hackl G, Eller P, et al. Impact of Sepsis on High-Density Lipoprotein Metabolism. Front Cell Dev Biol. 2021;9:795460.

13.    Samara I, Karikas GA, Kalkani E, Tzimogianni A, Bournousouzis N, Fytou-Pallikari A. Negative correlations of serum total-homocysteine and HDL-c levels in ICU patients. Health Science Journal. 2010;4(3):189–92.

14.    Schrutka L, Distelmaier K, Meyer B, Wurm R, Heinz G, Pacher R, et al. Impaired antioxidant high-density lipoprotein function predicts poor outcome in critically ill patients. Eur Heart J Acute Cardiovasc Care. 2015;4(SUPPL. 1):82.

15.    Sharma NK, Tashima AK, Brunialti MKC, Ferreira ER, Torquato RJS, Mortara RA, et al. Proteomic study revealed cellular assembly and lipid metabolism dysregulation in sepsis secondary to community-acquired pneumonia. Sci Rep. 2017 Dec 1;7(1).

16.    Sharma NK, Ferreira BL, Tashima AK, Brunialti MKC, Torquato RJS, Bafi A, et al. Lipid metabolism impairment in patients with sepsis secondary to hospital acquired pneumonia, a proteomic analysis. Clin Proteomics. 2019 Jul;16(1):29.

17.    Tanaka S, De Tymowski C, Zappella N, Snauwaert A, Robert T, Lortat-Jacob B, et al. Lipoprotein concentration in patients requiring extracorporeal membrane oxygenation. Scientific Reports 2021 11:1. 2021 Aug;11(1):1–10.

18.    Tsai MH, Peng YS, Chen YC, Lien JM, Tian YC, Fang JT, et al. Low serum concentration of apolipoprotein A-I is an indicator of poor prognosis in cirrhotic patients with severe sepsis. J Hepatol. 2009 May;50(5):906–15.

19.    Yamano S, Shimizu K, Ogura H, Hirose T, Hamasaki T, Shimazu T, et al. Low total cholesterol and high total bilirubin are associated with prognosis in patients with prolonged sepsis. J Crit Care. 2016 Feb;31(1):36–40.

**Supplementary Table 4**

Summarises differences in included studies between this meta-analysis and Hofmaenner et al 2023

| **Studies included by Hofmaenner et al which were not included in this meta-analysis** | **Reason for exclusion** |
| --- | --- |
| Sharma et al 2017 | Assessment of lipids not <24 hours ICU admission |
| Sharma et al 2019 | Assessment of lipids not <24 hours  ICU admission |
| Guirgis et al 2020 | Phase 1 interventional trial |
| Guirgis et al 2021 | Mixed cohort of intensive care and emergency department subjects, unable to access sufficient data to analyse only the intensive care cohort |
| Yamano et al 2016 | Retrospective cohort (assessed retrospectively subjects with duration of ICU stay >2 weeks) |
| **Studies included in this meta-analysis that were not included by Hofmaenner et al** | **Brief summary of study (cohort type and number of subjects)** |
| Bonville et al 2004 | SIRS cohort (n=109) |
| Chien et al 2015 | Severe community acquired pneumonia cohort (n=40) |
| Desmoulin et al 2013 | Acute heart failure cohort (n=126) |
| Etogo Asse et al 2013 | Acute liver failure cohort (n=163) |
| Fraunberger et al 2000 | Multi organ failure cohort (n= 33) |
| Guimaraes et al 2008 | Acute kidney injury cohort (n=56) |
| Inal et al 2015 | Sepsis cohort (n=85) |
| Oliva et al 2022 | Sepsis cohort (n = 224) |
| Peng et al 2015 | Cohort with predicted severe acute pancreatitis (n=66) |
| Reisinger et al 2020 | Cohort with sepsis and non sepsis control cohort (n=78) |
| Tachyla et al 2017 | Sepsis cohort with multiorgan failure (n=67) |
| Uzundere et al 2015 | General ICU cohort (n=502) |
