# Supplementary material for: Low circulatory levels of total cholesterol, HDL-C and LDL-C are associated with death of patients with sepsis and critical illness: systematic review, meta-analysis, and perspective of observational studies
Source: eBioMedicine. 2024 Jan 29;100:104981. doi: 10.1016/j.ebiom.2024.104981 (PMC10844818; doi:10.1016/j.ebiom.2024.104981)

Supplementary Figure 1

	Non-Survivor			Survivor			
**Author(s) and Year**	Mean	SD	N	Mean	SD	N	**MD [95% CI]**
Barlage et al 2009	81.07	38.47	47	94.77	38.45	104	-13.70 [-26.95, -0.45]
Berbee et al 2008	88.55	55.98	8	96.49	93.49	9	-7.94 [-80.30, 64.42]
Bonville et al. 2004	140.8	16.5	27	133.1	4.9	82	7.70 [1.39, 14.01]
Delirrad et al. 2020	139	20.9	56	140.89	26.92	32	-1.89 [-12.70, 8.92]
Desmoulin at al. 2013	91.65	25.91	28	124.9	39.06	98	-33.25 [-45.58, -20.92]
Etogo-Asse et al. 2012	65.09	35.19	64	81.84	43.64	99	-16.75 [-28.92, -4.58]
Guimaraes et al. 2008	80.3	35.7	39	147.4	53.1	17	-67.10 [-94.72, -39.48]
Khaliq et al 2020	50.27	15.08	8	110.36	47.95	11	-60.09 [-90.29, -29.89]
Lee et al 2015	90.5	54.9	52	105.33	40.94	65	-14.83 [-32.77, 3.11]
Lekkou et al 2014	118.1	41.2	22	129.8	34.3	28	-11.70 [-33.10, 9.70]
Memis et al 2007	92.2	25.1	41	175.1	38.6	55	-82.90 [-95.67, -70.13]
Peng at al. 2015	106	48.17	13	122	45.72	53	-16.00 [-44.93, 12.93]
Reisinger et al. 2020	105	41.22	29	128	50.51	49	-23.00 [-43.62, -2.38]
Tachyla et al. 2017	148.53	97.7	29	145.37	40.76	38	3.16 [-34.69, 41.01]
Tanaka et al 2017	67.67	33.56	12	96.16	46.64	63	-28.49 [-50.70, -6.28]
Tanaka et al. 2020	119.88	59.72	16	126.32	45.01	32	-6.44 [-39.60, 26.72]
Tanaka et al. 2022	76.57	29.39	42	95.13	34.42	184	-18.56 [-28.75, -8.37]
Uzundere et al 2015	122.1	41.4	172	129.2	38.8	330	-7.10 [-14.57, 0.37]

RE Model (Q = 166.84, df = 15, I^2 =91.5%)

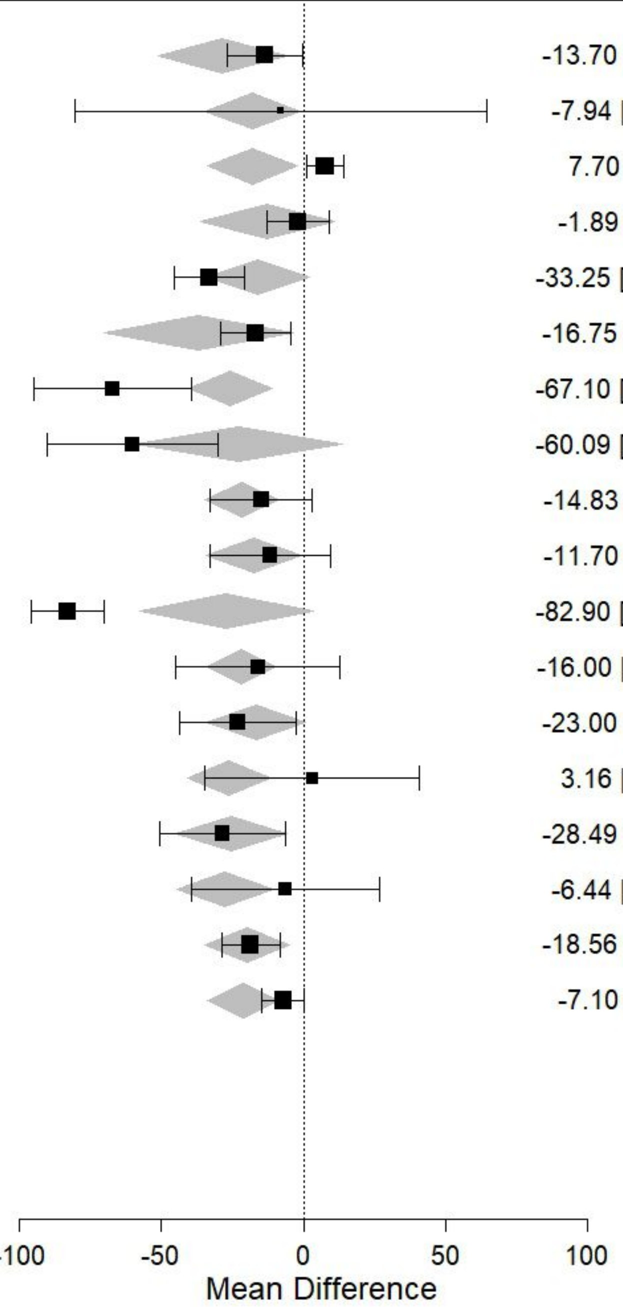

Supplementary Figure 2

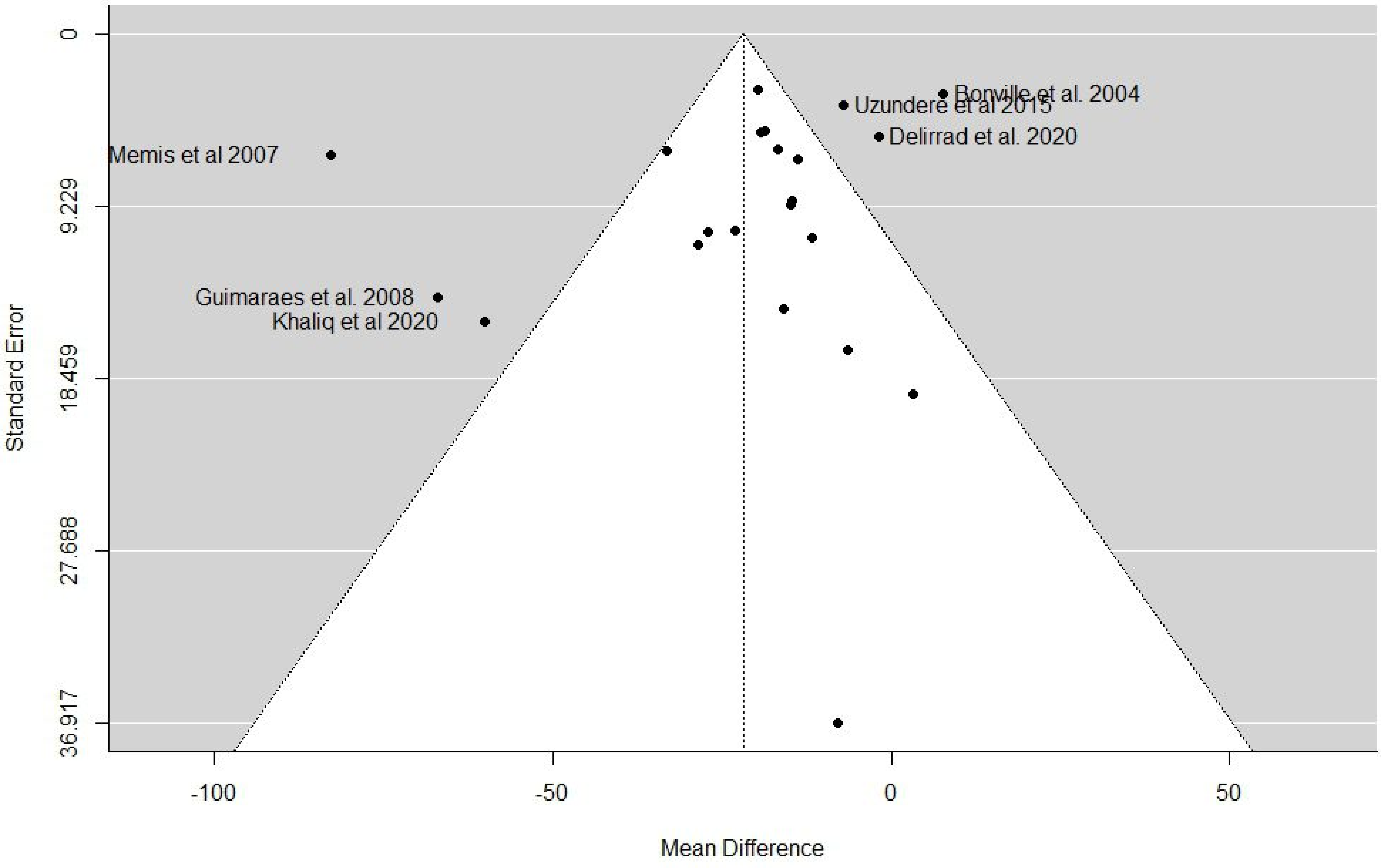

Supplementary Figure 3

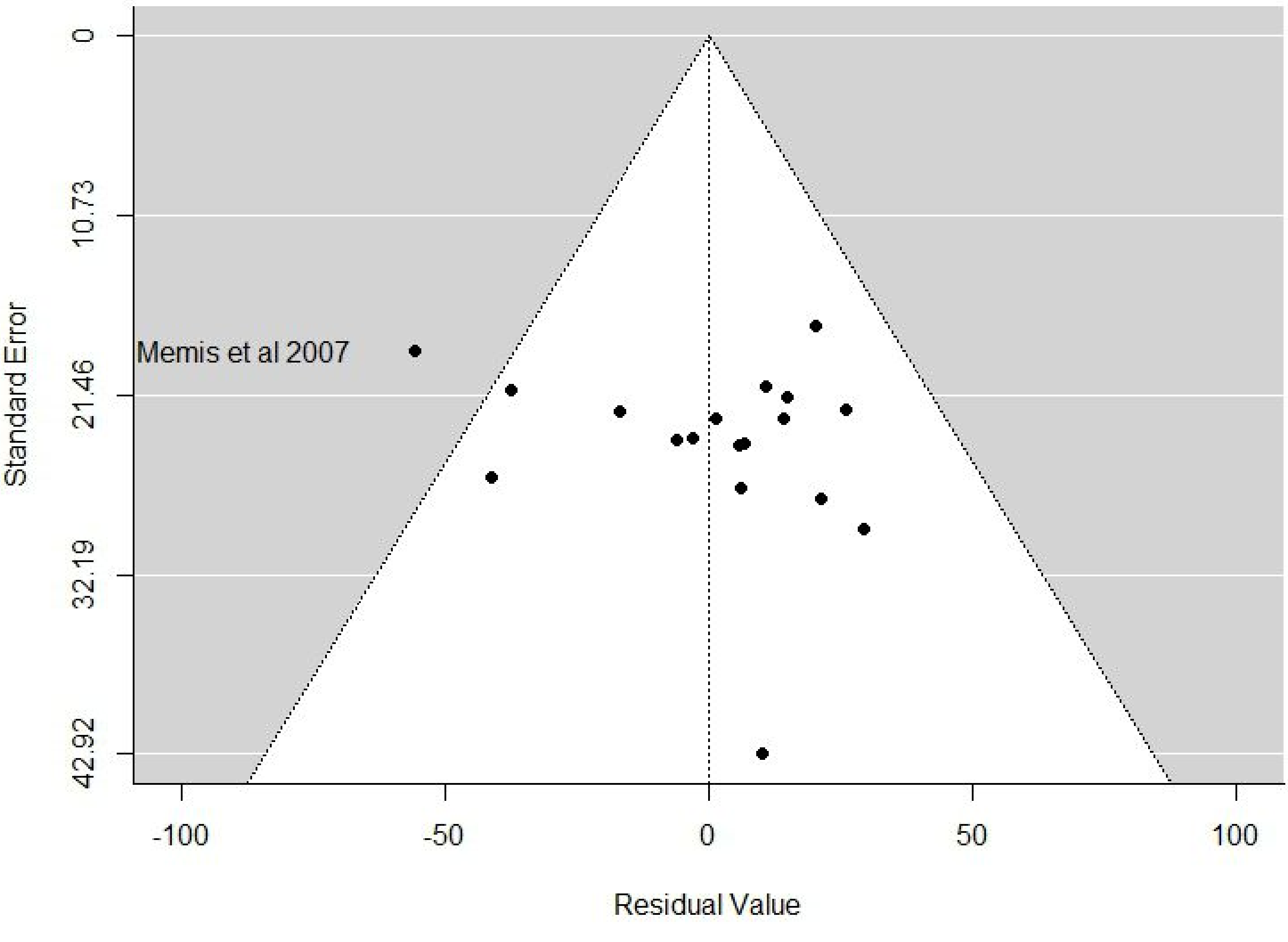

Supplementary Figure 4

Author(s) and Year	Non-Survivor Mean	Non-Survivor SD	Non-Survivor N	Survivor Mean	Survivor SD	Survivor N	MD [95% CI]
Barlage et al 2009	81.07	38.47	47	94.77	38.45	104	-13.70 [-26.95, -0.45]
Berbee et al 2008	88.55	55.98	8	96.49	93.49	9	-7.94 [-80.30, 64.42]
Bonville et al. 2004	140.8	16.5	27	133.1	4.9	82	7.70 [1.39, 14.01]
Delirrad et al. 2020	139	20.9	56	140.89	26.92	32	-1.89 [-12.70, 8.92]
Desmoulin at al. 2013	91.65	25.91	28	124.9	39.06	98	-33.25 [-45.58, -20.92]
Etogo-Asse et al. 2012	65.09	35.19	64	81.84	43.64	99	-16.75 [-28.92, -4.58]
Fraunberger et al. 2000	73	24	20	100	33	13	-27.00 [-47.79, -6.21]
Guimaraes et al. 2008	80.3	35.7	39	147.4	53.1	17	-67.10 [-94.72, -39.48]
Guirgis et al. 2018	90.6	30.69	15	105.3	34.2	67	-14.70 [-32.26, 2.86]
Khaliq et al 2020	50.27	15.08	8	110.36	47.95	11	-60.09 [-90.29, -29.89]
Lee et al 2015	90.5	54.9	52	105.33	40.94	65	-14.83 [-32.77, 3.11]
Lekkou et al 2014	118.1	41.2	22	129.8	34.3	28	-11.70 [-33.10, 9.70]
Levels et al 2007	47.56	7.73	11	67.29	7.35	15	-19.73 [-25.62, -13.84]
Oliva et al 2022	135.34	38.67	92	154.68	38.67	132	-19.34 [-29.63, -9.05]
Peng at al. 2015	106	48.17	13	122	45.72	53	-16.00 [-44.93, 12.93]
Reisinger et al. 2020	105	41.22	29	128	50.51	49	-23.00 [-43.62, -2.38]
Tachyla et al. 2017	148.53	97.7	29	145.37	40.76	38	3.16 [-34.69, 41.01]
Tanaka et al 2017	67.67	33.56	12	96.16	46.64	63	-28.49 [-50.70, -6.28]
Tanaka et al. 2020	119.88	59.72	16	126.32	45.01	32	-6.44 [-39.60, 26.72]
Tanaka et al. 2022	76.57	29.39	42	95.13	34.42	184	-18.56 [-28.75, -8.37]
Uzundere et al 2015	122.1	41.4	172	129.2	38.8	330	-7.10 [-14.57, 0.37]
lodel (Q = 95.64, df = 20, I^2 =77.9%)							-17.63 [-24.23, -11.02]

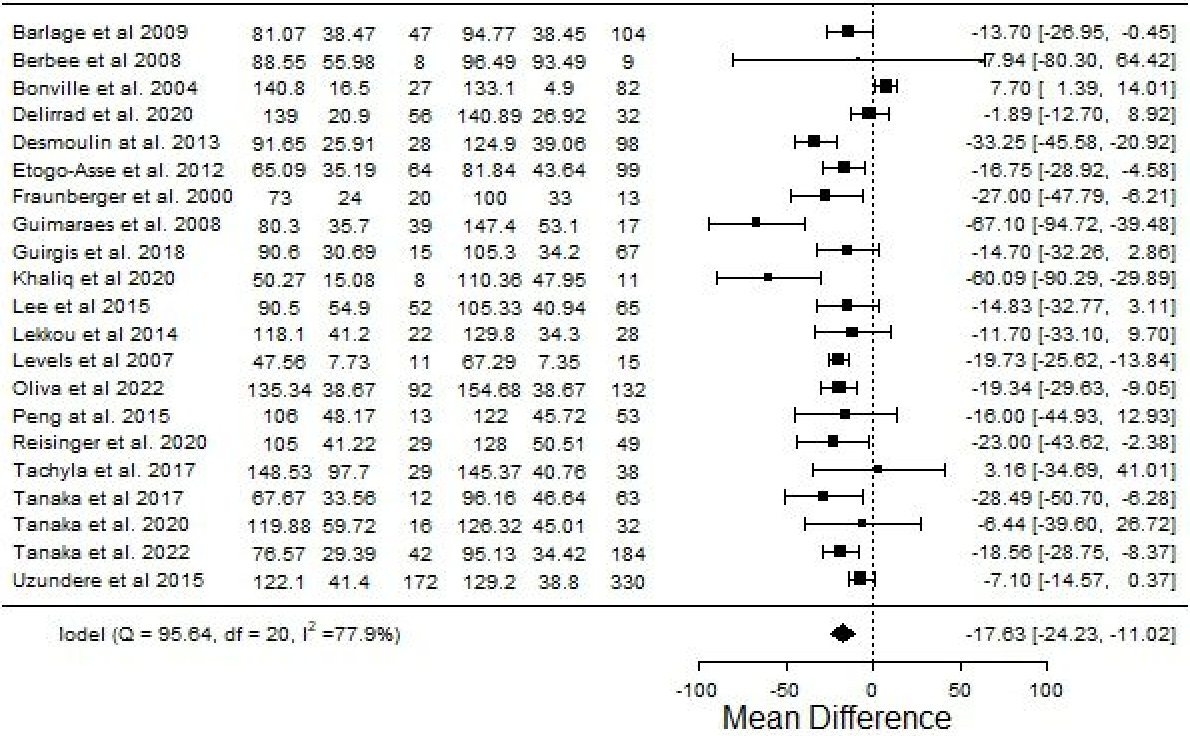

Supplementary Figure 5

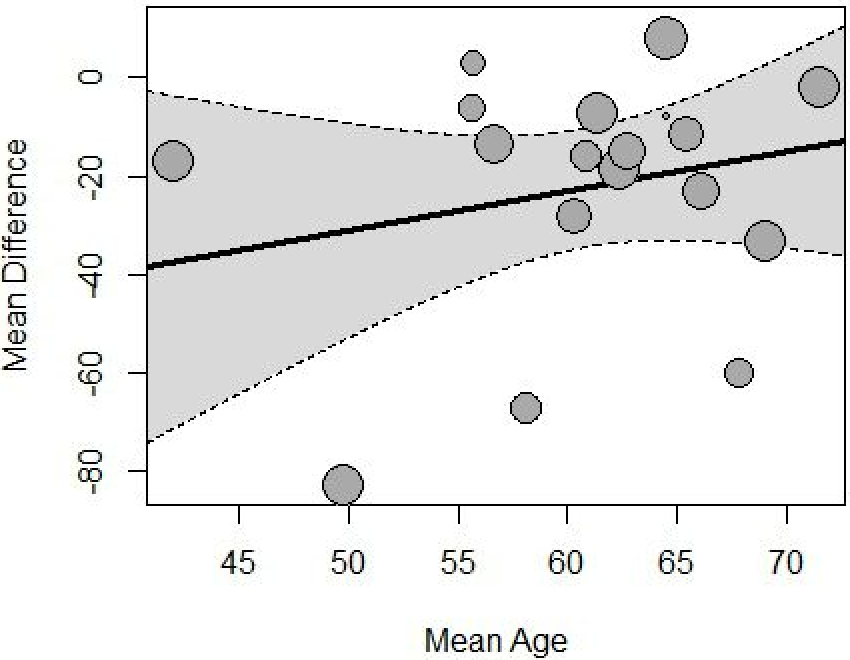

Supplementary Figure 6

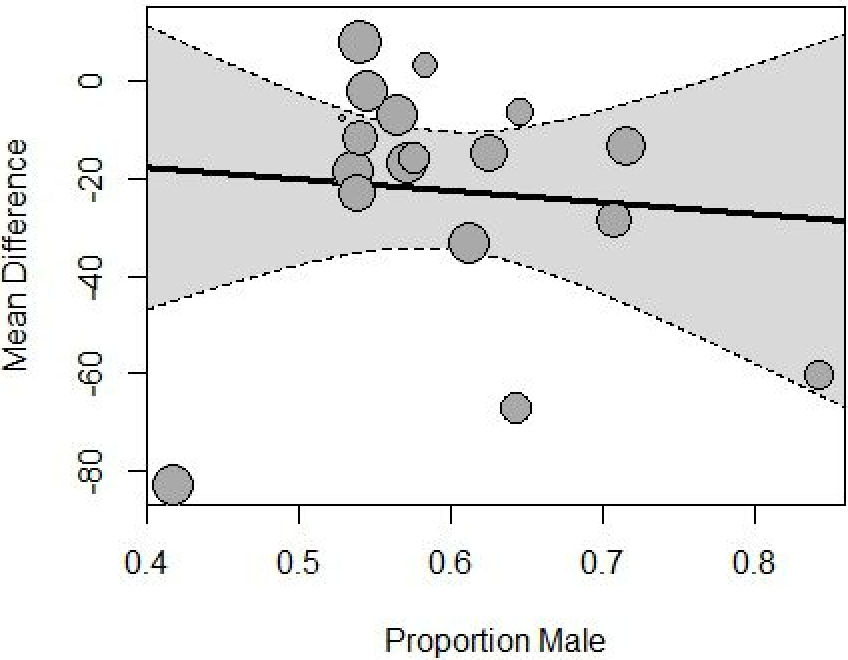

Supplementary Figure 7

Author(s) and Year	Non-Survivor Mean	Non-Survivor SD	Non-Survivor N	Survivor Mean	Survivor SD	Survivor N	MD [95% CI]
Barlage et al 2009	81.07	38.47	47	94.77	38.45	104	-13.70 [-26.95, -0.45]
Berbee et al 2008	88.55	55.98	8	96.49	93.49	9	-7.94 [-80.30, 64.42]
Bonville et al. 2004	140.8	16.5	27	133.1	4.9	82	7.70 [1.39, 14.01]
Delirrad et al. 2020	139	20.9	56	140.89	26.92	32	-1.89 [-12.70, 8.92]
Desmoulin at al. 2013	91.65	25.91	28	124.9	39.06	98	-33.25 [-45.58, -20.92]
Etogo-Asse et al. 2012	65.09	35.19	64	81.84	43.64	99	-16.75 [-28.92, -4.58]
Guimaraes et al. 2008	80.3	35.7	39	147.4	53.1	17	-67.10 [-94.72, -39.48]
Khaliq et al 2020	50.27	15.08	8	110.36	47.95	11	-60.09 [-90.29, -29.89]
Lee et al 2015	90.5	54.9	52	105.33	40.94	65	-14.83 [-32.77, 3.11]
Lekkou et al 2014	118.1	41.2	22	129.8	34.3	28	-11.70 [-33.10, 9.70]
Peng at al. 2015	106	48.17	13	122	45.72	53	-16.00 [-44.93, 12.93]
Reisinger et al. 2020	105	41.22	29	128	50.51	49	-23.00 [-43.62, -2.38]
Tachyla et al. 2017	148.53	97.7	29	145.37	40.76	38	3.16 [-34.69, 41.01]
Tanaka et al 2017	67.67	33.56	12	96.16	46.64	63	-28.49 [-50.70, -6.28]
Tanaka et al. 2020	119.88	59.72	16	126.32	45.01	32	-6.44 [-39.60, 26.72]
Tanaka et al. 2022	76.57	29.39	42	95.13	34.42	184	-18.56 [-28.75, -8.37]
Uzundere et al 2015	122.1	41.4	172	129.2	38.8	330	-7.10 [-14.57, 0.37]

RE Model (Q = 55.89, df = 14, I^2 =75%)

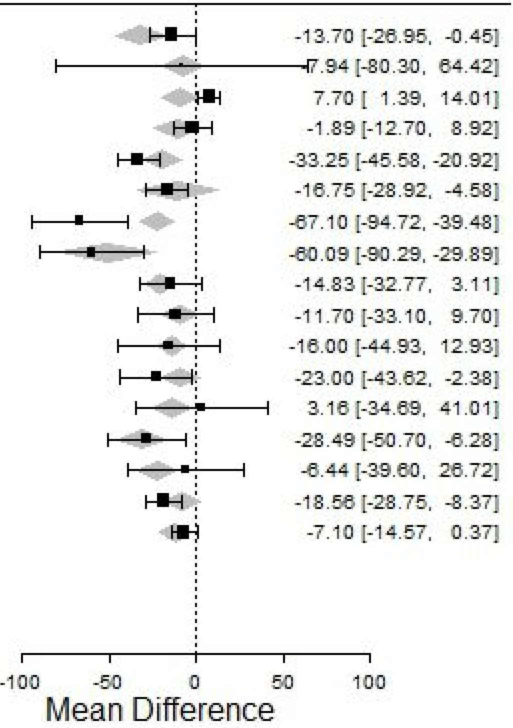

Supplementary Figure 8

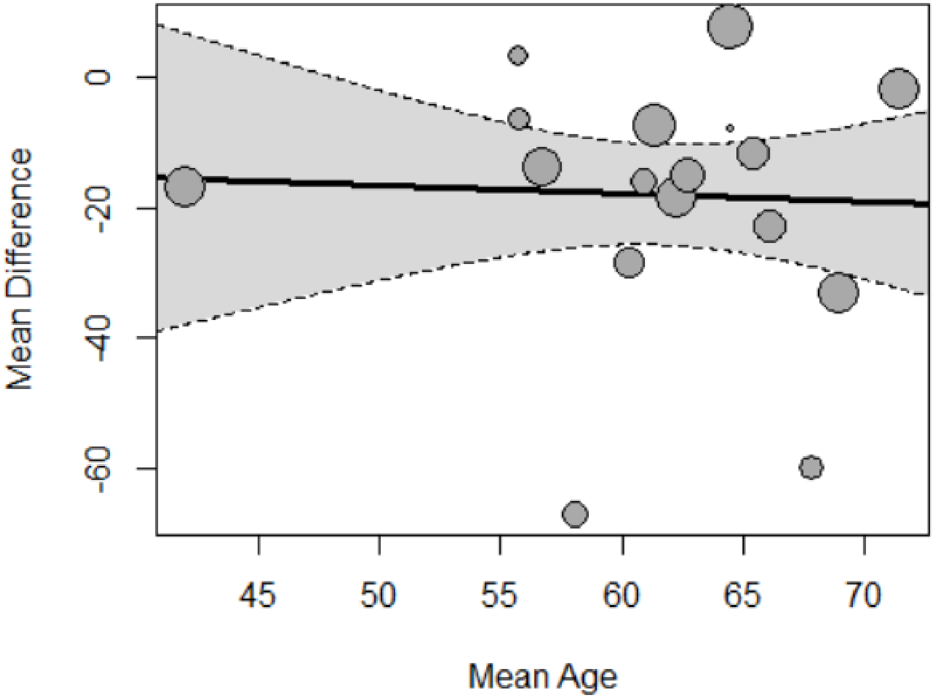

Supplementary Figure 9

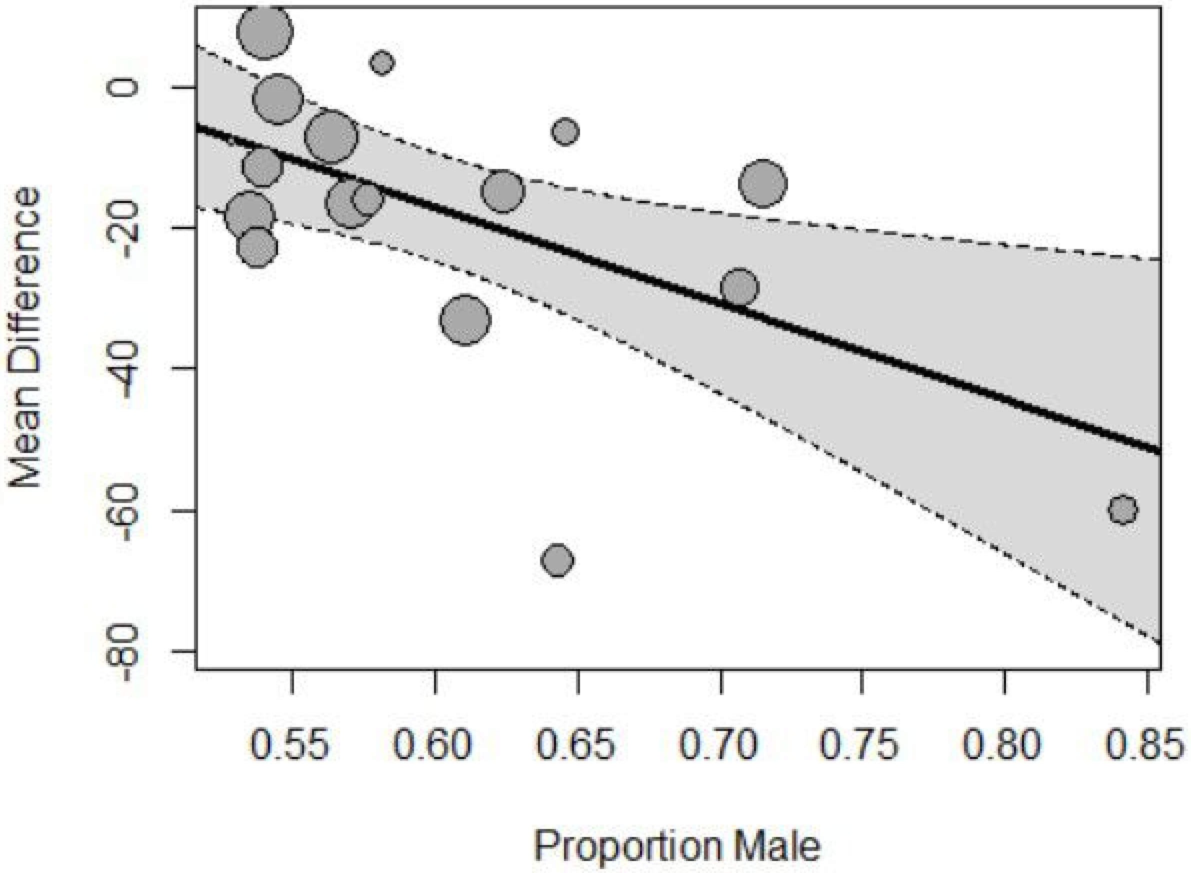

Supplementary Figure 10

Author(s) and Year	Non-Survivor Mean	Non-Survivor SD	Non-Survivor N	Survivor Mean	Survivor SD	Survivor N	MD [95% CI]
Barlage et al 2009	10.69	10.06	47	14.95	12.5	104	-4.26 [-8.01, -0.51]
Berbee et al 2008	24.36	6.92	8	23.72	19.28	9	0.64 [-12.84, 14.12]
Bonville et al. 2004	27.3	4	27	32.3	1.8	82	-5.00 [-6.56, -3.44]
Delirrad et al. 2020	36.25	8	56	36.88	10.2	32	-0.63 [-4.74, 3.48]
Desmoulin at al. 2013	35	9	28	47	15	98	-12.00 [-16.46, -7.54]
Etogo-Asse et al. 2012	7.86	11.43	64	18.05	11.64	99	-10.19 [-13.81, -6.57]
Khaliq et al 2020	7.73	2.73	8	27.84	19.56	11	-20.11 [-31.82, -8.40]
Lee et al 2015	19	13.72	52	21.5	13.27	65	-2.50 [-7.43, 2.43]
Lekkou et al 2014	18.8	7.6	22	28	8.2	28	-9.20 [-13.59, -4.81]
Peng at al. 2015	7.67	7.475	13	19.67	15.24	53	-12.00 [-17.77, -6.23]
Reisinger et al. 2020	21	15.54	29	32	22.46	49	-11.00 [-19.46, -2.54]
Tanaka et al 2017	13.85	14.43	12	25.52	20.83	63	-11.67 [-21.32, -2.02]
Tanaka et al. 2020	24.49	12.57	16	29.65	18	32	-5.16 [-13.93, 3.61]
Tanaka et al. 2022	18.56	11.21	42	21.27	13.15	184	-2.71 [-6.60, 1.18]
Chien at al. 2015	27.1	17.2	15	35.1	20.2	25	-8.00 [-19.77, 3.77]
Inal et al 2015	34.5	8.9	39	43.6	8.1	46	-9.10 [-12.74, -5.46]

RE Model (Q = 39.46, df = 13, I^2 =71.9%)

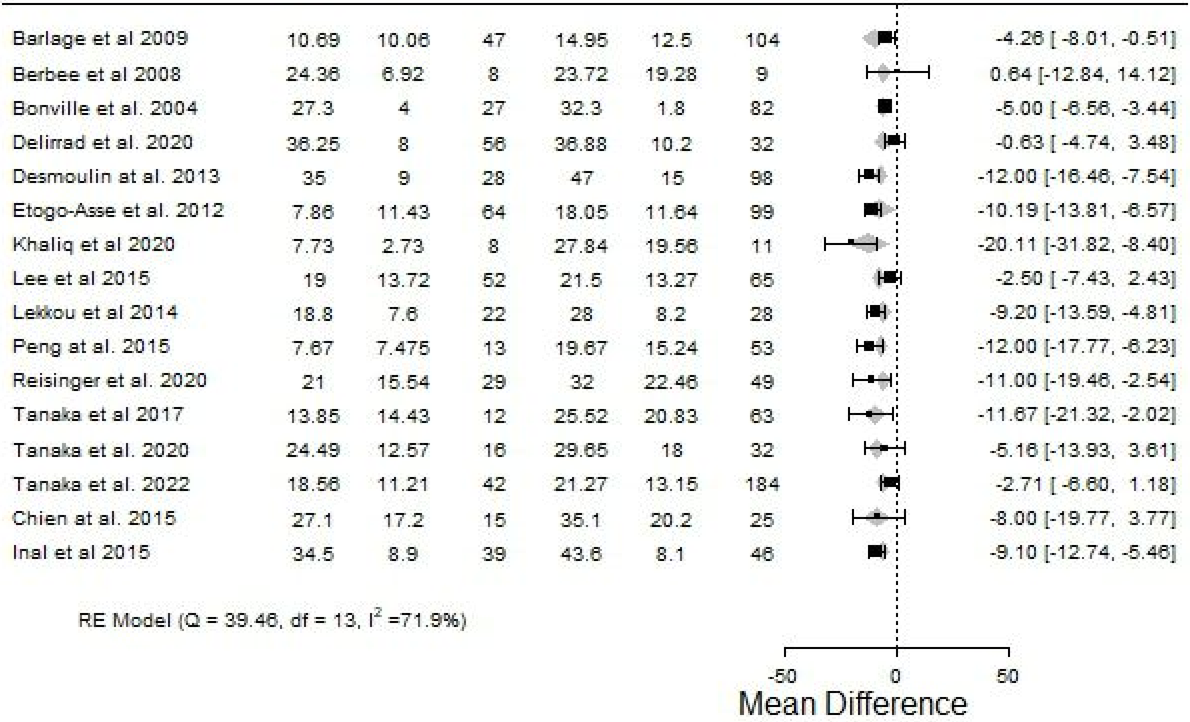

Supplementary Figure 11

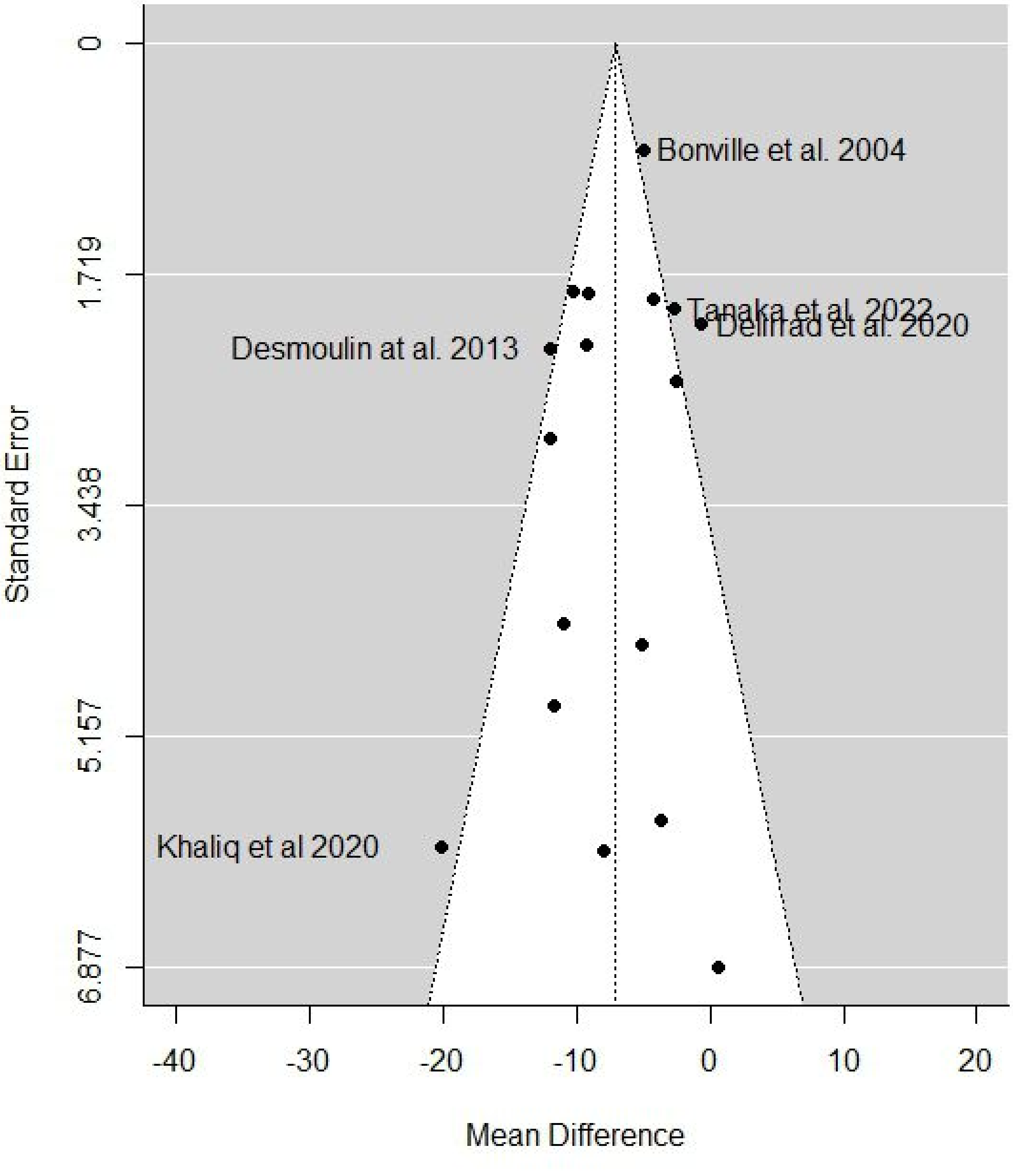

Supplementary Figure 12

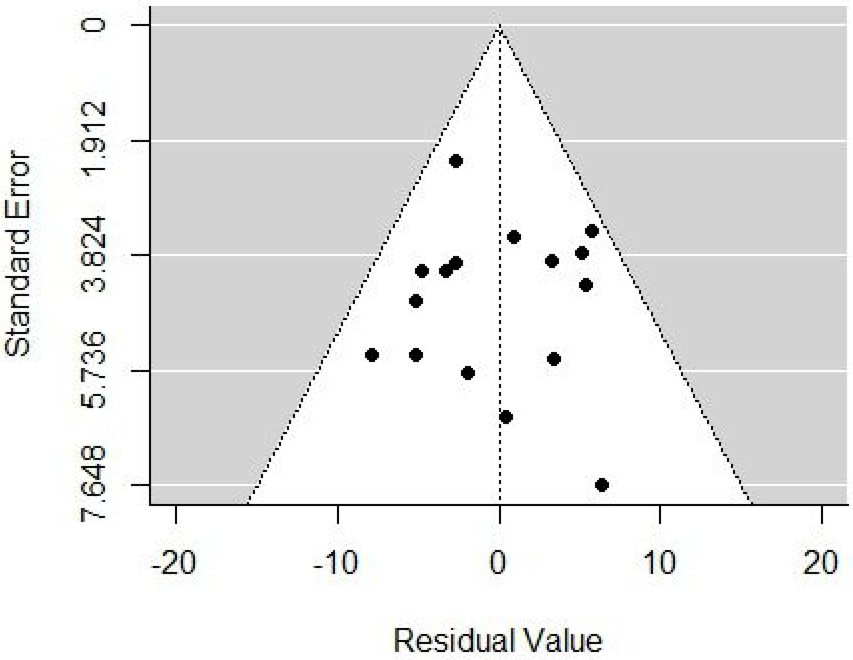

Supplementary Figure 13

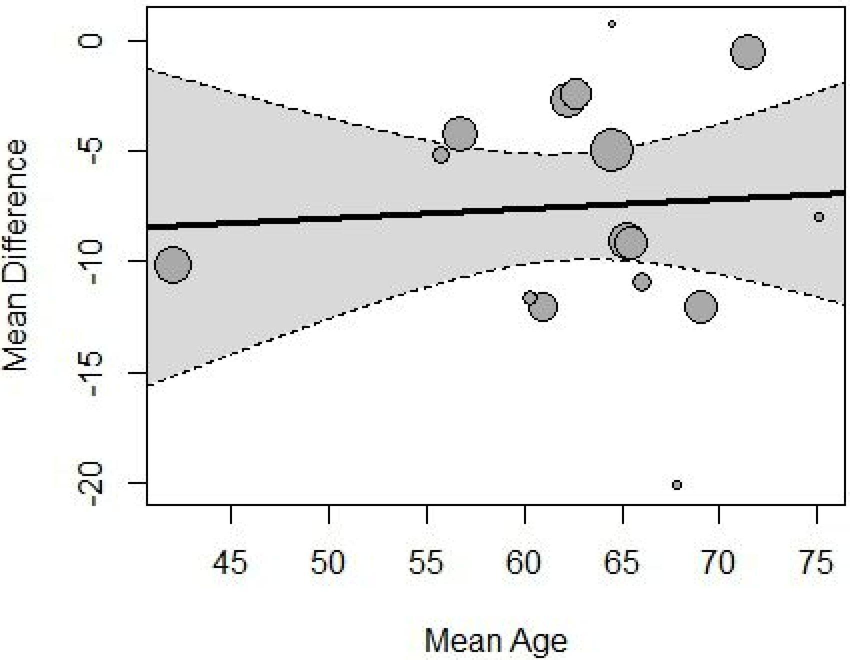

Supplementary Figure 14

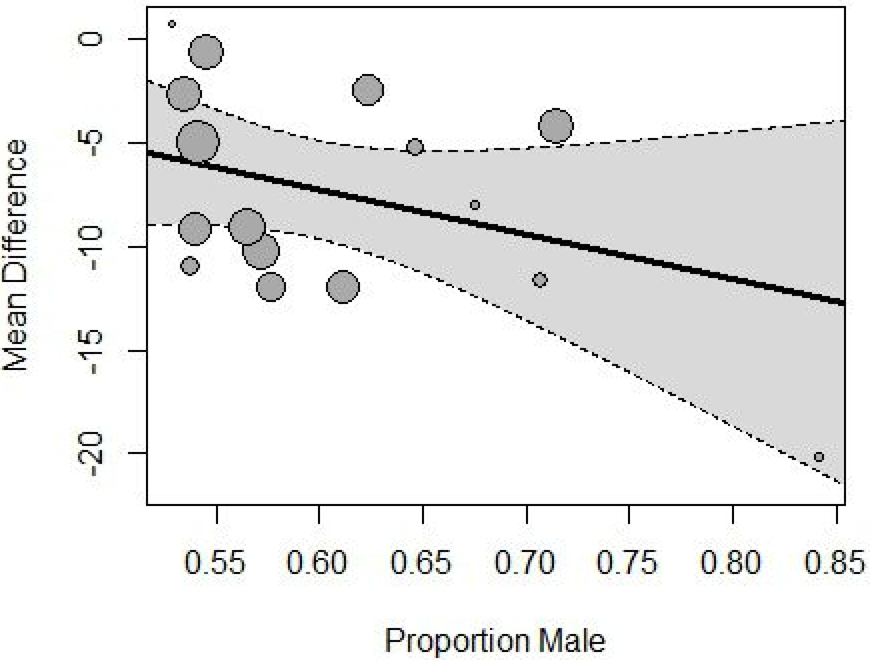

Supplementary Figure 15

	Non-Survivor			Survivor			
**Author(s) and Year**	Mean	SD	N	Mean	SD	N	**MD [95% CI]**
Barlage et al 2009	31.71	28.98	47	43.95	23.55	104	-12.24 [-21.68, -2.80]
Berbee et al 2008	40.86	11.75	8	28.48	37.21	9	12.38 [-13.26, 38.02]
Bonville et al. 2004	75.9	10.6	27	73.3	4	82	2.60 [-1.49, 6.69]
Delirrad et al. 2020	79.28	18.53	56	77.37	19.28	32	1.91 [-6.35, 10.17]
Desmoulin at al. 2013	84	36	28	108	43	98	-24.00 [-39.82, -8.18]
Etogo-Asse et al. 2012	39.96	26.4	64	41.25	29.09	99	-1.29 [-9.93, 7.35]
Khaliq et al 2020	20.88	10.44	8	62.26	41.58	11	-41.38 [-66.99, -15.77]
Lee et al 2015	42	32.79	52	48.33	30.33	65	-6.33 [-17.90, 5.24]
Lekkou et al 2014	67.3	39.5	22	75.8	31.9	28	-8.50 [-28.80, 11.80]
Peng at al. 2015	54.67	50.67	13	60	29.71	53	-5.33 [-34.01, 23.35]
Tanaka et al 2017	34.32	35.1	12	46.73	30.8	63	-12.41 [-33.68, 8.86]
Tanaka et al. 2020	61.87	34.57	16	73.47	30.01	32	-11.60 [-31.48, 8.28]
Tanaka et al. 2022	32.1	24.75	39	44.47	27.46	172	-12.37 [-21.16, -3.58]
Chien at al. 2015	37.9	29.3	15	47.5	30.6	25	-9.60 [-28.67, 9.47]

RE Model (Q = 18.28, df = 11, I^2 =44.3%)

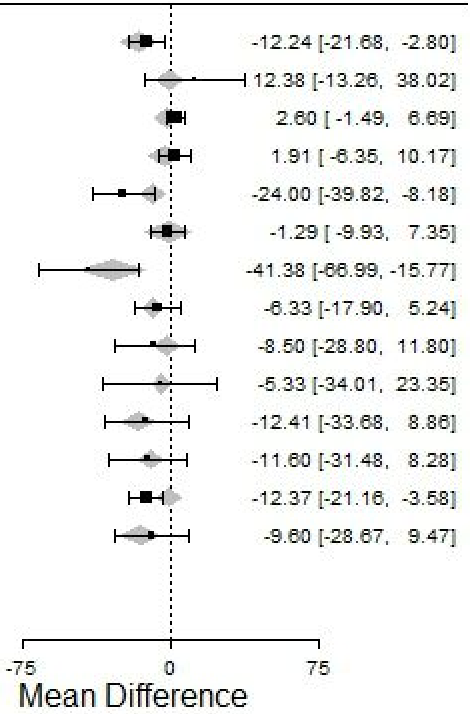

Supplementary Figure 16

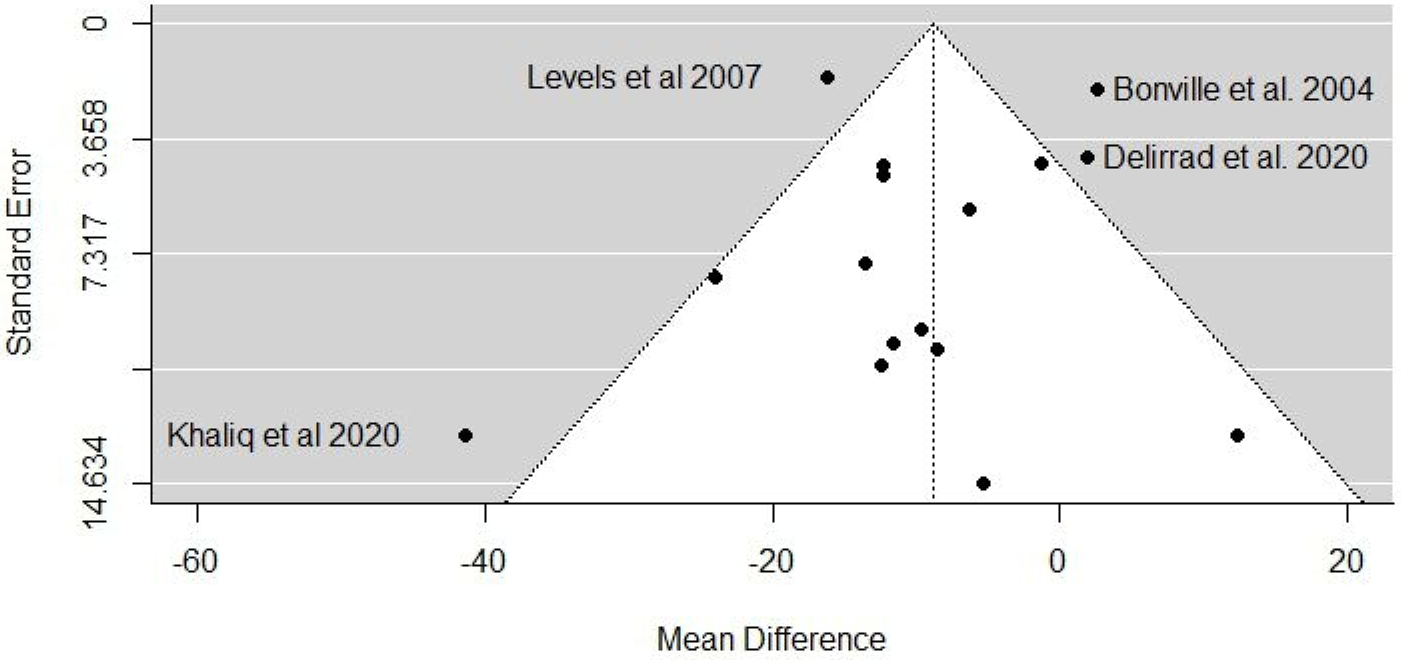

Supplementary Figure 17

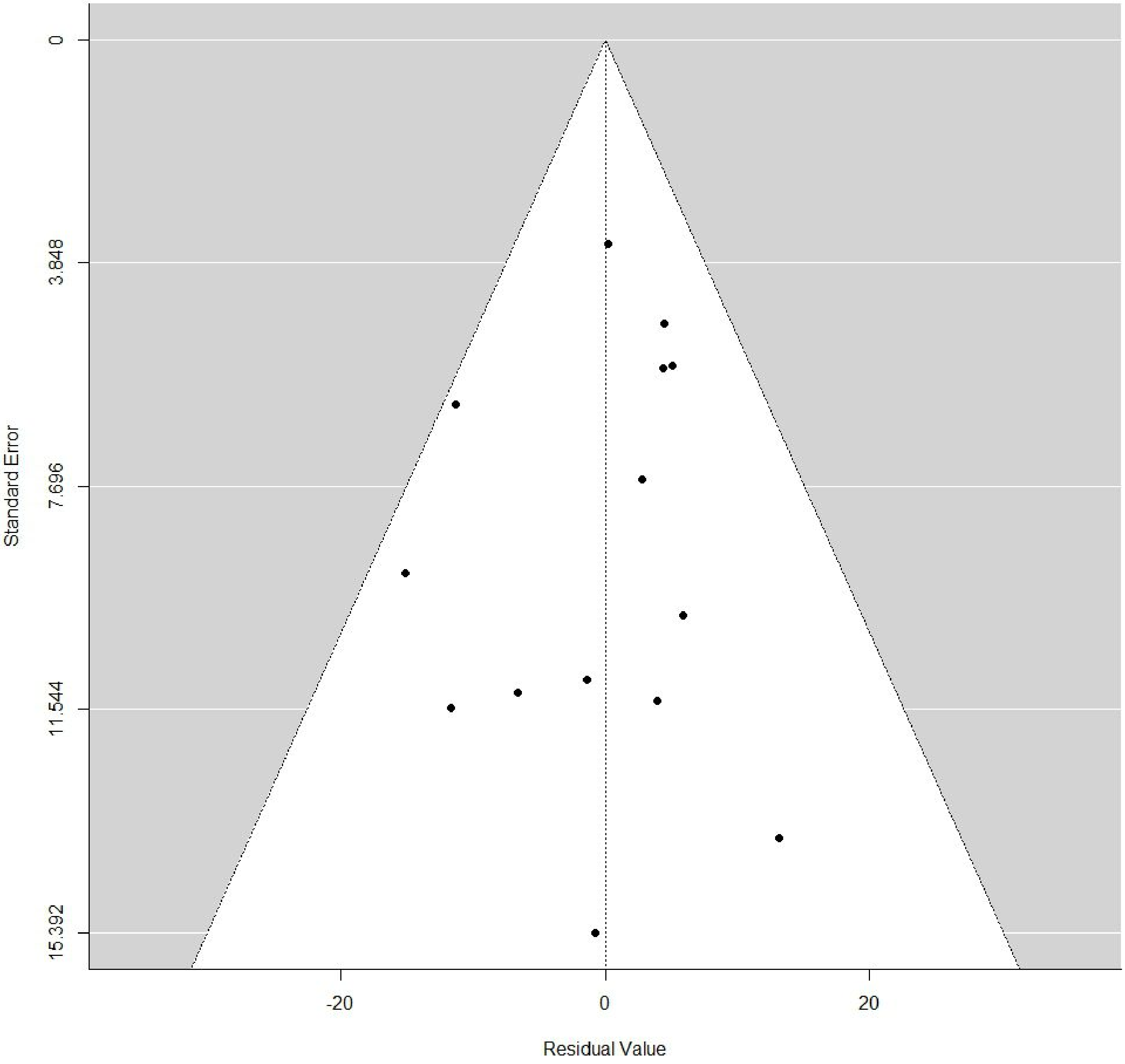

Supplementary Figure 18

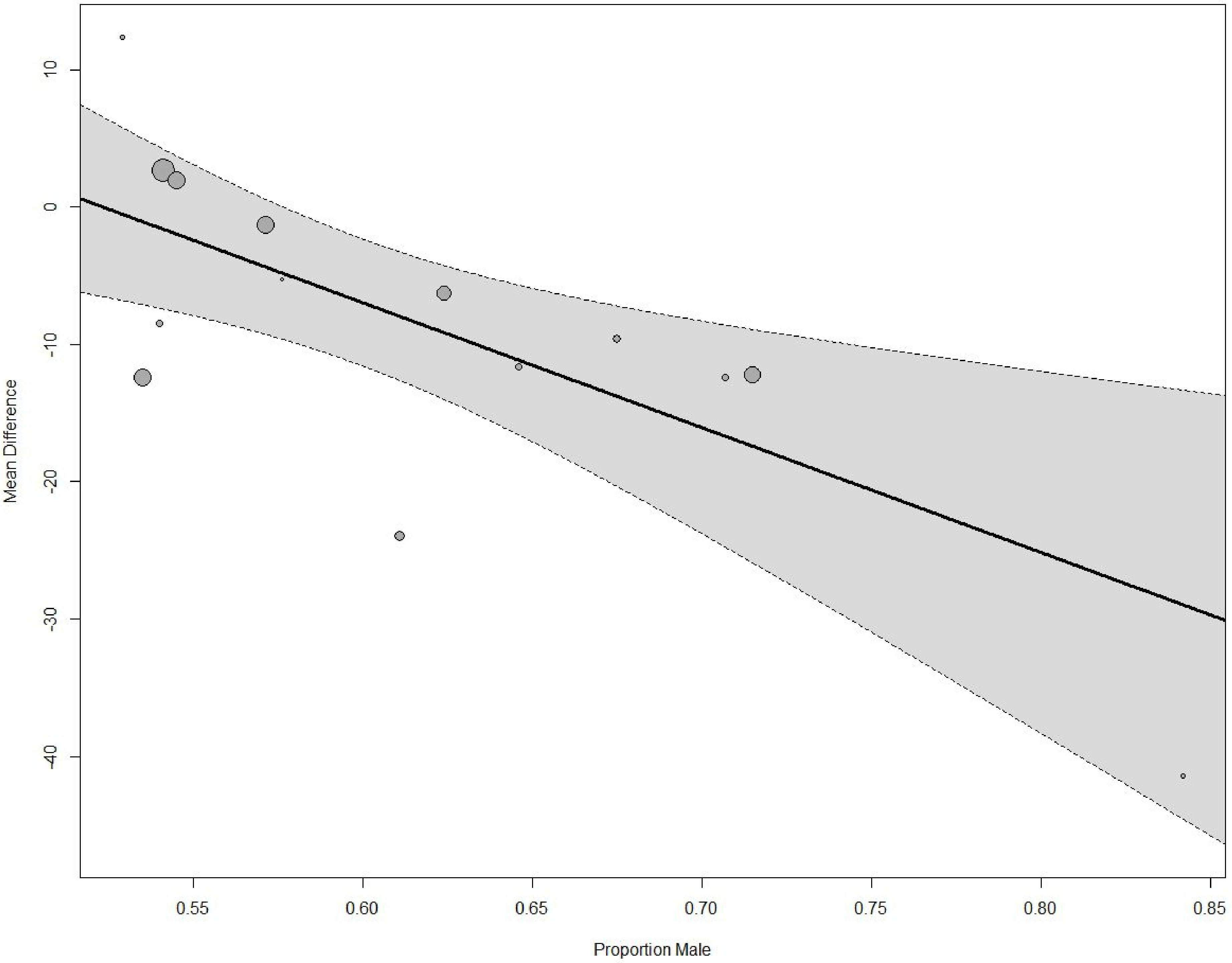

Supplementary Figure 19

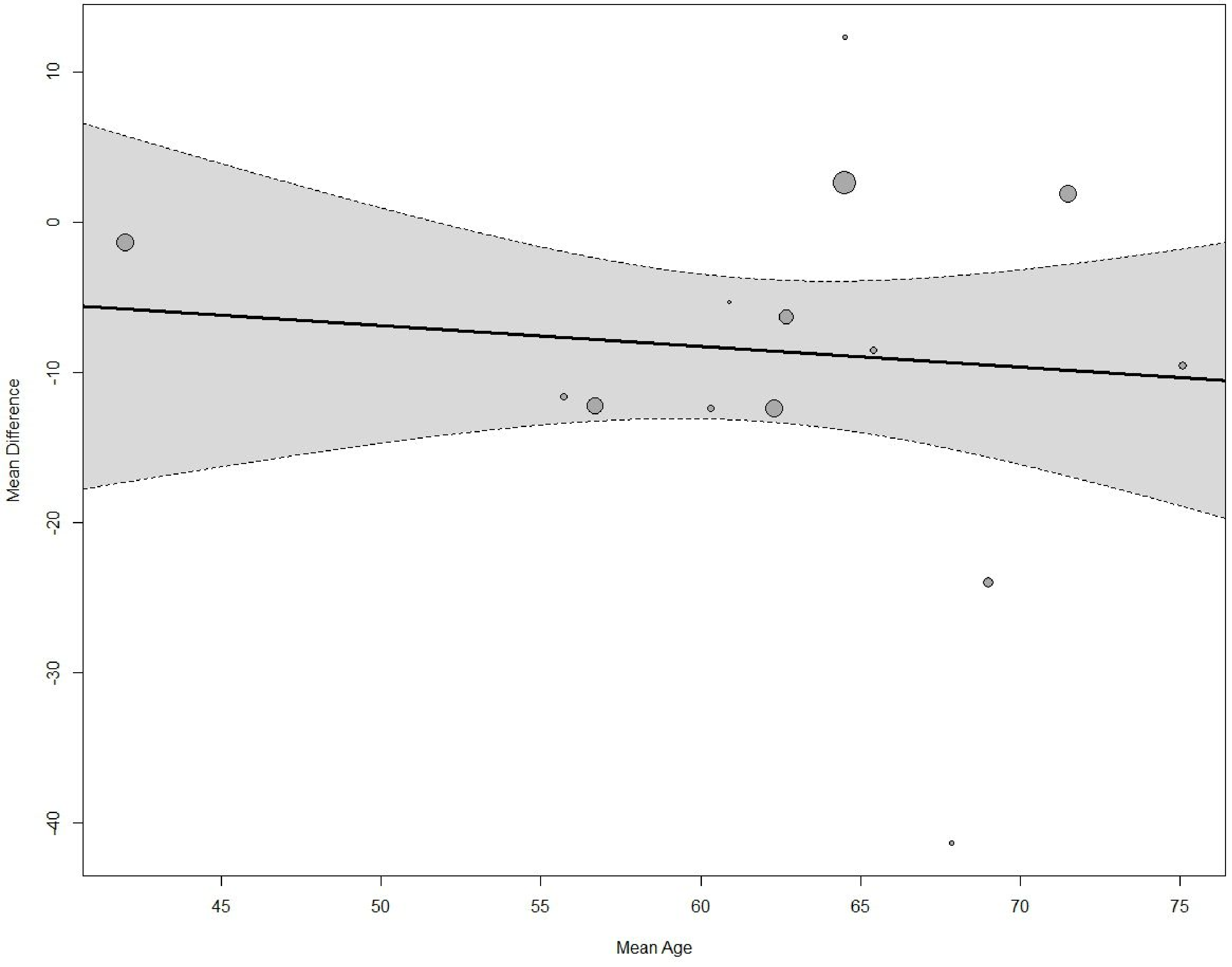

Supplement: Supplementary Figs. S1–S19 — Supplementary Fig. 1: adjusted meta-analysis for total cholesterol showing mean difference in serum total cholesterol between non-survivors and survivors (control) across studies (mg/dL) adjusted for mean age and proportion male subjects for studies which had this data available in the manuscript. Heterogeneity shown as I2. Supplementary Fig. 2: funnel plot for unadjusted Total Cholesterol meta-analysis showing study standard error plotted against mean difference between survivor and non-survivor groups for day 1 ICU total cholesterol. Supplementary Fig. 3: funnel plot for adjusted Total Cholesterol meta-analysis showing study standard error plotted against mean difference between survivor and non-survivor groups for day 1 ICU total cholesterol. Supplementary Fig. 4: unadjusted meta-analysis for total cholesterol, with Memis et al excluded, showing pooled mean difference in serum total cholesterol and 95% CI between non-survivors and survivors (control) across studies (mg/dL). Heterogeneity shown as I2. Supplementary Fig. 5: meta-regression for total cholesterol showing effect of mean age (years) as a covariable. Shaded area indicates the 95% confidence band. Supplementary Fig. 6: meta-regression for total cholesterol, with showing effect of sex (proportion male) as a covariable. Shaded area indicates the 95% confidence band. Supplementary Fig. 7: adjusted meta-analysis for total cholesterol, without Memis et al, showing mean difference in serum total cholesterol between non-survivors and survivors (control) across studies (mg/dL) adjusted for mean age and proportion male subjects for studies which had this data available in the manuscript. Heterogeneity shown as I2. Supplementary Fig. 8: meta-regression for total cholesterol, without Memis et al, showing effect of mean age (years) as a covariable. Shaded area indicates the 95% confidence band. Supplementary Fig. 9: meta-regression for total cholesterol, without Memis et al, showing effect of sex (proportion male) a [file mmc2.pdf]
